# Habitat and land‐use intensity shape moth community structure across temperate forest and grassland

**DOI:** 10.1111/1365-2656.70132

**Published:** 2025-09-09

**Authors:** Rafael Achury, Michael Staab, Sebastian Seibold, Jörg Müller, Lea Heidrich, Marcel Püls, Hermann Hacker, Carlos Roberto Fonseca, Markus Fischer, Nico Blüthgen, Wolfgang Weisser

**Affiliations:** ^1^ Terrestrial Ecology Research Group, Department of Ecology and Ecosystem Management, School of Life Sciences Technische Universität München Freising Germany; ^2^ Ecological Networks Technische Universität Darmstadt Darmstadt Germany; ^3^ Animal Ecology and Trophic Interactions, Institute of Ecology Leuphana University Lüneburg Lüneburg Germany; ^4^ Forest Zoology Dresden University of Technology Tharandt Germany; ^5^ Bavarian Forest National Park Grafenau Germany; ^6^ Chair of Conservation Biology and Forest Ecology, Field Station Fabrikschleichach University of Würzburg Rauhenebrach Germany; ^7^ Environmental Informatics, Faculty of Geography Philipps‐Universität Marburg Marburg Germany; ^8^ Departamento de Ecologia Universidade Federal do Rio Grande do Norte Natal Brazil; ^9^ Institute of Plant Sciences University of Bern Bern Switzerland

**Keywords:** alpha‐ and beta diversity, artificial light at night (ALAN), coverage‐based diversity, forest and grassland heterogeneity, Lepidoptera, light trapping, moth diversity, plant diversity

## Abstract

1. Land‐use change and intensification are major drivers of biodiversity loss, yet their effects on diversity have usually been studied within a single habitat type or land‐use category, limiting our understanding of cross‐habitat patterns. Moths, a species‐rich taxon worldwide, represent a significant portion of the biodiversity in both temperate forests and grasslands, functioning as pollinators and herbivores. While increasing land‐use intensity (LUI) in both habitats is expected to negatively impact moth assemblages, the strength of this effect remains uncertain. Moreover, land‐use intensification interacts with broader environmental factors, such as weather conditions and the spread of artificial light at night (ALAN), but their combined effects on moth community diversity and turnover across habitats remain poorly understood.
2. We sampled moth communities across 150 grassland and 150 forest plots along land‐use gradients in Germany. We quantified plot‐ and landscape‐scale LUI and tested the role of plant diversity, temperature and precipitation during the night of sampling and the preceding season, and ALAN in shaping moth diversity (standardized by coverage) along Hill numbers.
3. Forests supported significantly higher moth abundance, biomass and diversity than grasslands, with habitat type being the main driver of moth community composition. LUI at the plot scale had contrasting effects on moth abundance, increasing it in forests but reducing it in grasslands. Impacts of LUI were more pronounced at the landscape level, reducing moth diversity particularly in areas dominated by grasslands. Plant diversity and temperature were key determinants for moth communities, increasing alpha diversity across diversity metrics, that is Hill numbers. ALAN had no significant influence on moth abundance or biomass but significantly decreased Simpson diversity. Beta diversity increased with geographic distance, habitat change and LUI but decreased with weather differences among plots.
4. Our results highlight the interplay between LUI, habitat type and abiotic factors in shaping moth communities across large spatial scales. Effective conservation strategies should consider maintaining habitat heterogeneity and promoting plant diversity, particularly in temperate habitats exposed to high land‐use intensification.

## Graphical abstract

This study reveals how land‐use intensity, plant diversity, weather and artificial light interact to shape moth diversity across forests and grasslands. Using 300 plots across Germany, we highlight habitat‐specific responses and beta‐diversity patterns, providing novel cross‐habitat insights at an unprecedented spatial scale for temperate nocturnal insect communities.

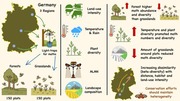

## Zusammenfassung

1. Landnutzungsänderung und ‐intensivierung sind zentrale Treiber des Biodiversitätsverlustes. Ihre Auswirkungen auf die Diversität wurden jedoch meist für bestimmte Habitattypen oder Landnutzungskategorien untersucht, was unser Verständnis über habitatübergreifende Muster einschränkt. Nachtfalter, eine weltweit artenreiche Gruppe, stellen einen bedeutenden Teil der Biodiversität sowohl in temperaten Wäldern als auch in Grünlandhabitaten dar und erfüllen Funktionen als Bestäuber und Herbivore. Obwohl eine zunehmende Landnutzungsintensität sowohl im Grünland als auch im Wald voraussichtlich negative Effekte auf Nachtfaltergemeinschaften hat, bleibt die Stärke dieses Effekts ungewiss. Zudem interagiert Landnutzungsintensivierung mit Umweltfaktoren wie Wetterbedingungen oder künstlicher Beleuchtung.
2. Wir untersuchten Nachtfaltergemeinschaften auf jeweils 150 Grünland‐ und 150 Waldflächen entlang von Landnutzungsgradienten in Deutschland. Wir quantifizierten die Landnutzungsintensität auf Plot‐ und Landschaftsebene und testeten die Rolle von Pflanzendiversität, von Temperatur und Niederschlag zum Zeitpunkt der Beprobung und in der vorausgehenden Saison, und von künstlicher Beleuchtung auf die Nachtfalterdiversität.
3. Wälder wiesen eine signifikant höhere Abundanz, Biomasse und Diversität von Nachtfaltern auf als Grünländer, wobei der Habitattyp der wichtigste Faktor für die Zusammensetzung der Gemeinschaft war. Die Landnutzungsintensität auf Plotskala zeigte gegensätzliche Effekte auf die Abundanz von Nachtfaltern: sie erhöhte die Diversität in Wäldern, verringerte sie aber im Grünland. Auf Landschaftsebene waren die Auswirkungen der Landnutzungsintensität stärker ausgeprägt und führten insbesondere in grünlanddominierten Gebieten zu einer Verringerung der Diversität. Pflanzendiversität und Temperatur waren Schlüsselfaktoren für Nachtfaltergemeinschaften und erhöhten die Alpha‐Diversität über verschiedene Diversitätsmaße (Hill‐Zahlen) hinweg. ALAN hatte keinen signifikanten Einfluss auf Abundanz oder Biomasse, verringerte jedoch die Simpson‐Diversität deutlich. Die Beta‐Diversität nahm mit geographischer Distanz, Habitatwechsel und Landnutzungsintensität zu, verringerte sich jedoch bei zunehmenden Wetterunterschieden zwischen den Flächen.
4. Unsere Ergebnisse verdeutlichen das Zusammenspiel von Landnutzungsintensität, Habitattyp und abiotischen Faktoren auf Nachtfaltergemeinschaften. Effektive Schutzstrategien sollten den Erhalt von Habitatheterogenität und die Förderung von Pflanzendiversität berücksichtigen, insbesondere in temperaten Habitaten mit hoher Landnutzungsintensität.

## 1 INTRODUCTION

Land‐use change and intensification are major drivers of biodiversity loss worldwide (Díaz et al., 2019; Jaureguiberry et al., 2022; Newbold et al., 2015), altering species interactions, ecosystem functions and community dynamics (Allan et al., 2015). These effects are evident in changes to animal communities across ecosystems. For instance, in temperate grasslands, arthropod species declined more strongly at sites with higher arable field cover over a 10‐year period (Seibold et al., 2019). Similarly, in temperate forests, intensive management reduces habitat heterogeneity and resource availability (Schall et al., 2018), contributing to insect biodiversity loss (Staab et al., 2023). However, most studies have focused on single habitats (but see Macgregor et al., 2019), limiting insights into whether land‐use drivers act consistently across ecosystems.

Beyond changes in biomass, abundance, and local (alpha) and regional (gamma) diversity, land‐use intensification can alter species composition (Flynn et al., 2009) by homogenizing communities (i.e. reduced beta diversity; Gossner et al., 2016). While alpha diversity remains the most common measure in biodiversity assessments, beta diversity, which incorporates species identities, has received less attention. Yet it may be more effective than alpha diversity for detecting changes across habitats rather than within single habitats (Müller et al., 2023). This is especially relevant where strong species turnover is expected across sharp boundaries, such as distinct land‐use types shaped by varying environmental and landscape‐level drivers (Mori et al., 2018). However, beta diversity across habitats remains underexplored, despite evidence that land‐use effects are often scale‐ and context‐dependent. For instance, semi‐natural grasslands may enhance diversity within a habitat but reduce beta diversity across habitats by promoting biotic homogenization when dominant in the landscape (Montràs‐Janer et al., 2024).

Among Lepidoptera, moths constitute a significant component of biodiversity in temperate grasslands and forests (van Nieukerken et al., 2011). They are species‐rich herbivores with a wide spectrum of feeding strategies, from specialists to generalists, and serve as a vital food source for many taxa, including birds and bats (Kolkert et al., 2020). However, moths are increasingly threatened by habitat change and intensification (New, 2023). For example, in agricultural landscapes, greater arable land cover correlates with declines in already threatened moth species (Merckx, Marini, et al., 2012). Similarly, in grasslands, land‐use intensity (LUI), comprising grazing, fertilization and mowing, significantly reduces moth abundance, richness and diversity (Mangels et al., 2017). In forests, moth communities are negatively affected by disturbances (Uhl et al., 2023), woodland fragmentation (Lintott et al., 2014) and forest size (Slade et al., 2013). A general decline in UK forest moths has been linked to habitat deterioration (Fox et al., 2014). In contrast, forest stand age positively influences community composition, with mixed‐age stands supporting higher beta and gamma diversity (Merckx, Feber, et al., 2012). Despite these findings, it remains unclear whether similar mechanisms are at play across ecosystems.

In addition to habitat and land‐use effects, abiotic conditions such as temperature and precipitation also play a critical role in shaping moth communities, operating across multiple temporal scales. For instance, short‐term weather conditions, such as temperature and rainfall during sampling nights, affect moth activity, dispersal and detectability (Hordley et al., 2023; Mutshinda et al., 2011). In contrast, seasonal weather conditions (e.g. winter temperature and growing‐season rainfall) can influence vegetation productivity, overwintering survival and larval development, with consequences for population dynamics (Hill et al., 2021), as demonstrated for insects more broadly (Seibold et al., 2019). Plant species richness also determines moth communities by enhancing resource availability and habitat complexity, supporting both generalist and specialist species (e.g. Kühne et al., 2022; Root et al., 2017; Tobisch et al., 2023; Tyler, 2020). In addition, artificial light at night (ALAN) is an emerging driver of nocturnal biodiversity loss (Sanders et al., 2021), disrupting light cycles, foraging and behaviour (Merckx et al., 2023), and degrading habitat quality (van Grunsven et al., 2020; van Langevelde et al., 2017). These variables often interact in complex ways (Sanetra et al., 2024), making it critical to disentangle their individual and combined effects on moth diversity across spatial scales.

Here, we analysed the effects of land use and other key environmental drivers that can interact with land‐use change on moth communities in temperate landscapes. Using a spatially well‐replicated sampling design across 300 grassland and forest sites along gradients of LUI in Germany (Fischer et al., 2010), we examined the individual and combined effects of plot‐ and landscape‐scale land use, while also accounting for additional variables such as plant species richness, short‐ and seasonal‐scale weather conditions, and ALAN on moth abundance, biomass and diversity. To capture complementary aspects of diversity, we quantified diversity as well as compositional turnover (beta) along the Hill numbers, using the effective number of species, focusing on rare (q0), common (q1) and dominant species (q2) across habitats. Specifically, we addressed the following questions: (1) How are the abundance and biomass of moths at the plot level influenced by habitat type, LUI and weather conditions during sampling? (2) How do seasonal weather conditions, plant species richness, habitat, and LUI at both plot and landscape levels affect the alpha diversity of moth communities? (3) Are variations in moth species composition driven by geographical distance and/or environmental variables?

## 2 METHODS

### 2.1 Study system

This study was conducted within the Biodiversity Exploratories, a large‐scale, long‐term project investigating how land use affects biodiversity and related ecosystem functions and services (Fischer et al., 2010; www.biodiversity‐exploratories.de). Sampling plots are located in three regions: the UNESCO Biosphere Reserve Schorfheide‐Chorin in northeast Germany (52°47′25′′–53°13′26′′ N, 13°23′27′′–14°08′53′′ E, 3–140 m a.s.l.); the Hainich National Park and surrounding Hainich‐Dün area in central Germany (50°56′14′′–51°22′43′′ N, 10°10′24′′–10°46′45′′ E, 285–550 m a.s.l.); and the UNESCO Biosphere Reserve Swabian Alb in southwest Germany (48°20′28′′–48°32′02′′ N, 9°10′49′′–09°35′54′′ E, 460–860 m a.s.l.). Mean annual precipitation (MAP) ranges from 700 to 1000 mm in the Swabian Alb, 500–800 mm in Hainich‐Dün and 520–580 mm in Schorfheide‐Chorin. Mean annual temperature increases from Swabian Alb (6.0°C–7.0°C) to Hainich‐Dün (6.5°C–8.0°C) to Schorfheide‐Chorin (8.0°C–8.5°C).

In each region, 50 grassland plots (50 m × 50 m) and 50 forest plots (100 m × 100 m) were selected to span the full range of land‐use intensities typical of each region and habitat (see Fischer et al., 2010; Figure S1). Grasslands are actively managed as meadows (mown), pastures (grazed) or mown pastures (both), and may be fertilized or unfertilized. Forest plots include unmanaged deciduous stands, beech (*Fagus sylvatica* L.)‐dominated stands and age‐class forests with either European beech or a conifer as the dominant species. Conifer stands are dominated by Norway spruce [*Picea abies* (L.) H.Karst; Swabian Alb and Hainich‐Dün] or Scots pine (*Pinus sylvestris* L.; Schorfheide‐Chorin).

### 2.2 Moth sampling, biomass measurement and identification

To assess the effects of land use on nocturnal moths in grasslands and forests, semi‐automated light traps were used. Each trap consisted of a 12 V battery powering a 15 W ultraviolet fluorescent tube (Figure S2) with a twilight sensor that automatically switched the light on at dusk and off at dawn. A 10‐L plastic bucket with an inserted funnel was attached below, containing a screw‐cap vessel filled with chloroform and egg boxes for moths to hide. Rain shields were installed above the tube to protect the trap. Moths attracted to the light were killed by chloroform and collected the next day. Traps were emptied, and moths were transferred to a plastic box, stored in a cooler and frozen in mobile freezers at the end of the day.

In grasslands, traps were hung on the enclosure of the weather stations that are installed at every plot, while in forests, traps were deployed in the centre of each plot to optimally reflect the surrounding forest structure. Sampling was carried out in two periods within the peak of average moth phenology: first from 5 to 21 June 2018, and second from 9 July to 8 August 2018. Each plot was sampled twice to account for phenological variation in species composition and diversity (Summerville & Crist, 2002). All samplings were performed around the new moon phase (always ±1 week), to minimize moonlight interference. Within each round, plots per region were sampled over two to three nights (~39 traps per night on average) to reduce confounding weather effects. In the laboratory, total biomass per trap was measured using a precision balance (Mettler Toledo AB265‐S) with a plastic cup. Only macromoths (Lepidoptera: Macroheterocera) were identified (Weisser et al., 2021) based on morphology and genitalia. All specimens are stored at the University of Marburg. Field work permits were issued by the responsible state environmental offices of Baden‐Württemberg, Thüringen and Brandenburg.

### 2.3 LUI measures

#### 2.3.1 Plot‐scale land‐use

In grasslands, LUI is assessed annually via farmer questionnaires (Vogt et al., 2019). Mowing intensity was measured as the number of cutting events per year. Grazing intensity was represented by the standardized livestock units (cattle, sheep and/or horses) per hectare, times the number of days the plots were grazed per year. Fertilization intensity was quantified as nitrogen amounts from chemical fertilizer, manure or slurry per hectare. The land‐use intensities of the three land‐use components, that is mowing, grazing and fertilization, were summarized into a standardized index of LUI (see Blüthgen et al., 2012 for a more detailed description). For this analysis, values of 2018 of the land‐use questionnaire were used and standardized by the global mean (i.e. across all three regions) (Ostrowski et al., 2020).

For forests, LUI is based on forest inventories that were conducted on all plots between 2015 and 2018. All living trees with a diameter at breast height (DBH) greater than 7 cm were identified to species, and their DBH was recorded. Deadwood items (including stumps) with a diameter larger than 25 cm were measured (diameter and length) at the full plot. For smaller deadwood (7–25 cm diameter), measurements were taken along the diagonals of the site on two line transects, which were then extrapolated to cover 1 ha. From these data, a compound index (ForMIX) (Staab & Schall, 2025) that combines altered tree species composition, tree removal, deadwood availability and stand maturity, each in reference to natural old‐growth forest, was calculated (Staab et al., 2025).

Because LUI was measured differently in grasslands (LUI) and forests (ForMIX), we standardized (*z*‐transformed) LUI separately for forests and grasslands. The resulting scores were used as independent variables in statistical analyses of abundance and diversity for the joint analysis of forest and grassland plots (Table S1).

#### 2.3.2 Landscape‐scale land‐use

We quantified LUI at the landscape scale by calculating the proportion of area of different land‐use categories within circular areas centred around our plots. We selected a radius of 1000 m, as previous studies demonstrated that this scale captures the strongest landscape‐level effects on insect communities in our study regions, with biodiversity–land use correlations plateauing at larger scales (Seibold et al., 2019), and it explained most of the variation in macro‐moth diversity in arable lands of the UK (Merckx, Marini, et al., 2012). For land cover data, we utilized the 2018 ATKIS Basis DLM dataset (licence agreements: GeoBasis‐DE/LGB 2017, BG‐D 29/17), which provides polygon border accuracy within ±3 m. We focused on the percentages of grassland and arable land surrounding the plots for our analysis.

We also obtained data on the intensity of ALAN from the World Atlas of Artificial Night Sky Brightness, available at https://www.lightpollutionmap.info, using the zenith sky brightness in microcandela per square meter (μcd/m^2^) for each plot's coordinates. The data, with a spatial resolution of 15 arc seconds (i.e. precise for very small spatial areas) (Falchi et al., 2016), was used to quantify the degree of light pollution at each site.

### 2.4 Weather data

Air temperature (measured 2 m above the ground) data were recorded at all plots with hourly resolution for both 2017 and 2018. Any gaps in the time series for specific stations were filled using average linear relationships with nearby stations in the same regions (Wöllauer et al., 2025). Precipitation data were obtained from the RADOLAN product of the German Weather Service, which provides radar‐based precipitation estimates corrected by gauge measurements, with a resolution of 1 km^2^ and 0.1 mm/h. From these datasets, we calculated: (1) *Short‐term weather conditions*, with mean temperature and the total rainfall during the sampling event, which was roughly between 21:00 and 05:00 the next morning; and (2) *Seasonal weather conditions*, with mean winter temperature (from November of the previous year to February) and rainfall during the growing season (March to August).

### 2.5 Plant species diversity

Plant species diversity was calculated based on species cover estimates for each plot, with assessments conducted in both grasslands (Bolliger et al., 2020) and forests (Fischer et al., 2020) using slightly different methodologies (Boch et al., 2013; Socher et al., 2013). In grasslands, species cover was recorded in 4 m × 4 m relevés between mid‐May and mid‐June of 2018. In forests, species cover was measured in 20 m × 20 m plots, with assessments conducted twice during 2018 (in spring and summer). Using the species cover data from each plot, plant diversity was calculated as the exponential of the Shannon–Wiener Index (e.g. a measure of effective number of species). We then standardized (*z*‐transformed) the plant diversity values separately for each habitat type. These standardized scores were used as independent variables in statistical analyses of moth diversity, allowing for a unified analysis that integrated plant diversity across both grassland and forest habitats.

### 2.6 Statistical analyses

All statistical tests were conducted in R 4.3.3 (www.r‐project.org). Graphs were generated with the *ggplot2* package (v.3.5.1; Wickham, 2016). We employed generalized linear mixed‐effect models (GLMMs; using the *glmmTMB* package, v1.1.9; Brooks et al., 2017) across all analyses (except for beta diversity), to test the influence of various predictor variables on moth abundance, biomass, sample coverage and species diversity. All numerical fixed effects were scaled to mean = 0 and SD = ±1 to allow direct comparison of effect sizes. Error structures were adapted to each response variable: negative binomial for abundance, biomass and diversity; beta distribution for sample coverage.

#### 2.6.1 Analysis of moth abundances, biomass and sample coverage

We first examined whether moth abundance per trap‐night was influenced by habitat type (forest vs. grassland), plot‐scale LUI and their interaction (habitat × LUI), short‐term weather condition (i.e. mean temperature and total rainfall recorded between 21:00 and 05:00), ALAN, sampling period (June or July/August) and whether the trap was noted as having a problem during the morning inspection. We included the variable ‘Trap failure’ to account for cases where light traps failed during the inspection the following morning (99 of 600 trap‐nights), but it was often unclear whether failures occurred throughout the night or only in the morning. Traps were checked by manually covering the light sensor to verify whether the lamp activated. If the lamp did not turn on, indicating either low battery or other technical issues, we classified it as a failure. Despite a failure, it was common to find a high number of moths in the trap. While low abundances can lead to an underestimation of moth diversity within a plot, excluding data due to potential trap failures significantly reduces sampling size.

To assess whether low catches were associated with trap failure and might bias community patterns, we successively excluded plots with fewer than 5 (50/600 plots), 10 (64 plots), 15 (90 plots) and 20 (135 plots) individuals. We then evaluated how model results depended on the minimum number of individuals included in the analysis and found that trap failure was no longer significant when more than 10 individuals were captured (see Table S2; Supporting Information for additional discussion). To account for the hierarchical data structure with plots nested in regions, we specified a random intercept: Region/Plot (two measurements per plot). We repeated these analyses for moth biomass (see Table S3).

#### 2.6.2 Analysis of alpha diversity

For the subsequent diversity analyses, we pooled moth abundances per plot across the two sampling nights but excluded eight plots with fewer than 10 individuals, as these were strongly associated with trap failure (Table S2) and likely reflect technical errors rather than true moth activity patterns. One additional forest plot was excluded due to missing land use and covariate data. In total, 291 plots were analysed: 143 in grasslands and 148 in forests. Using the *iNEXT* package (version 3.0.1; Hsieh et al., 2022), we calculated sample coverage and moth diversity per plot using the rarefaction/extrapolation framework from Chao et al. (2014).

We estimated moth diversity at 70% and 90% sample coverage to account for differences in sampling completeness between plots, especially important when comparing habitats like grasslands and forests, which differ in moth abundance and richness, and when using light traps, whose catches are highly sensitive to night conditions. Standardizing diversity estimates by coverage reduces potential biases arising from incomplete sampling, ensuring that comparisons reflect true differences in community diversity rather than artefacts of unequal sampling effort and efficiency (Chao & Jost, 2012). Moth species diversity was estimated using three metrics based on Hill numbers: (a) Species richness (*q* = 0), which weighs all species equally, emphasizing rare species; (b) Shannon entropy (*q* = 1), which weighs species according to their frequency, without favouring rare or dominant species; and (c) Simpson diversity (*q* = 2), which assigns more weight to dominant species (Jost, 2006). These diversity metrics, together with sample coverage, were used as response variables to examine whether they were influenced by habitat type, plot‐ and landscape‐scale LUI, seasonal weather conditions (i.e. mean winter temperature and vegetation period precipitation), ALAN and plant diversity.

Additionally, we explored the interactions between habitat and plot‐scale and landscape‐scale LUI, but these interactions were not significant in any of the diversity models. ‘Region’ was included as a random intercept. Despite some correlation among predictor variables (range: *ρ* = −0.52–0.46), all variance inflation factors (VIF) were below 2.7. Therefore, potential issues related to multicollinearity (James et al., 2014) are expected to be minimal (Figure S3).

#### 2.6.3 Analysis of beta diversity

We estimated moth beta diversity standardized by coverage along the series of Hill numbers, implemented in the iNEXT.beta3D package (version 1.0.2; Chao et al., 2023). The dissimilarity matrices for beta diversity were calculated based on the Sørensen index (*q* = 0), the Horn index (*q* = 1) and the Morisita–Horn index (*q* = 2). This approach allowed us to standardize comparisons of community composition changes (beta diversity) across different habitats and environmental gradients, ensuring that variations in sampling effort did not bias the results (Püls et al., 2025).

For each predictor variable (the same that we used for the alpha‐diversity analyses), and based on Euclidean distances between each pair of plots, we constructed dissimilarity matrices of size *N* × *N*, where *N* is the number of plots in our dataset. The predictor matrices were standardized before analysis (mean = 0, SD = 1; function *decostand* in vegan package; Oksanen et al., 2024), so their coefficients refer to a common scale, allowing for direct comparisons of their relative contributions to beta‐diversity patterns.

To investigate how moth community composition was affected by the variables that we also used for the alpha‐diversity analyses, we employed multiple regression on distance matrices (MRM) (Lichstein, 2007) using the *MRM* function from the ecodist package (version 2.1.3; Goslee & Urban, 2007). In addition, we used the coordinates of each plot to calculate a geographic distance matrix (using the *distm* function from the *geosphere* package; version 1.5‐20; Hijmans, 2024), which was included in the model to account for the tendency of areas that are closer to share more species than areas that are more distant (Dambros et al., 2020). Thus, MRM allowed us to test the relationship between moth species dissimilarity matrices and each predictor variable, after controlling for the effect of the other variables in the model. All models were performed with 1000 permutations of the response matrix. We also partitioned abundance‐based beta diversity into turnover and nestedness components using the *betapart* package (Baselga et al., 2023) for all plots and separately by habitat.

Finally, we used variance partitioning to examine the unique and shared contributions of predictor variables in explaining the variance in moth dissimilarity (modEvA package, version 3.20; Barbosa et al., 2013). The predictor variables were grouped into three categories: (1) Geographic/Weather (geographic distance, seasonal weather conditions); (2) Habitat (habitat type and plant diversity); and (3) Land use (LUI at plot and landscape scales, and ALAN). This was achieved by fitting reduced models (i.e. MRM models where one or more predictor variables were removed) using the same parameters as the full model and comparing the explained variance.

## 3 RESULTS

### 3.1 Trapping success

In total, 584 out of 600 trap‐nights provided data. During the 584 trap‐nights, we captured 71,388 individuals belonging to 455 macro‐moth species with an average of 122 individuals per trap‐night (range: 0–812 individuals). Forest plots averaged 197.9 ± 162.3 (mean ± SD) individuals per night, while grasslands averaged 45.9 ± 43.9. Across all forest plots and trap‐nights, 398 moth species were recorded (38.5 ± 17.7 species per trap‐night per plot). In grasslands, a total of 343 species were found, averaging 17.3 ± 11.3 species per trap‐night per plot. For forests, the number of individuals per trap‐night in the Alb was higher than in the Hainich and the Schorfheide. For grasslands, more individuals per trap‐night were captured in the Hainich than in the Schorfheide (Figure S4).

The most abundant species were *Campaea margaritaria* (4632 individuals, 266 plots), *Laspeyria flexula* (2470, 184) and *Lymantria monacha* (2345, 106) (Table S4). Forty‐eight species were represented by a single individual, and 31 by two. A total of 286 species (62.9%) were shared between forest and grassland, comprising 96.4% of all individuals. In contrast, 112 species (24.6%; 3.2% of individuals) were unique to forests, and 57 (12.5%; 0.4%) to grasslands (Figure S5). Overall, 189 species (41.5%) occurred across all three regions, with greater overlap between Hainich–Alb and Hainich–Schorfheide than between Alb–Schorfheide (Figure S6).

### 3.2 Moth abundance and biomass analyses (Q1)

**FIGURE 1:**
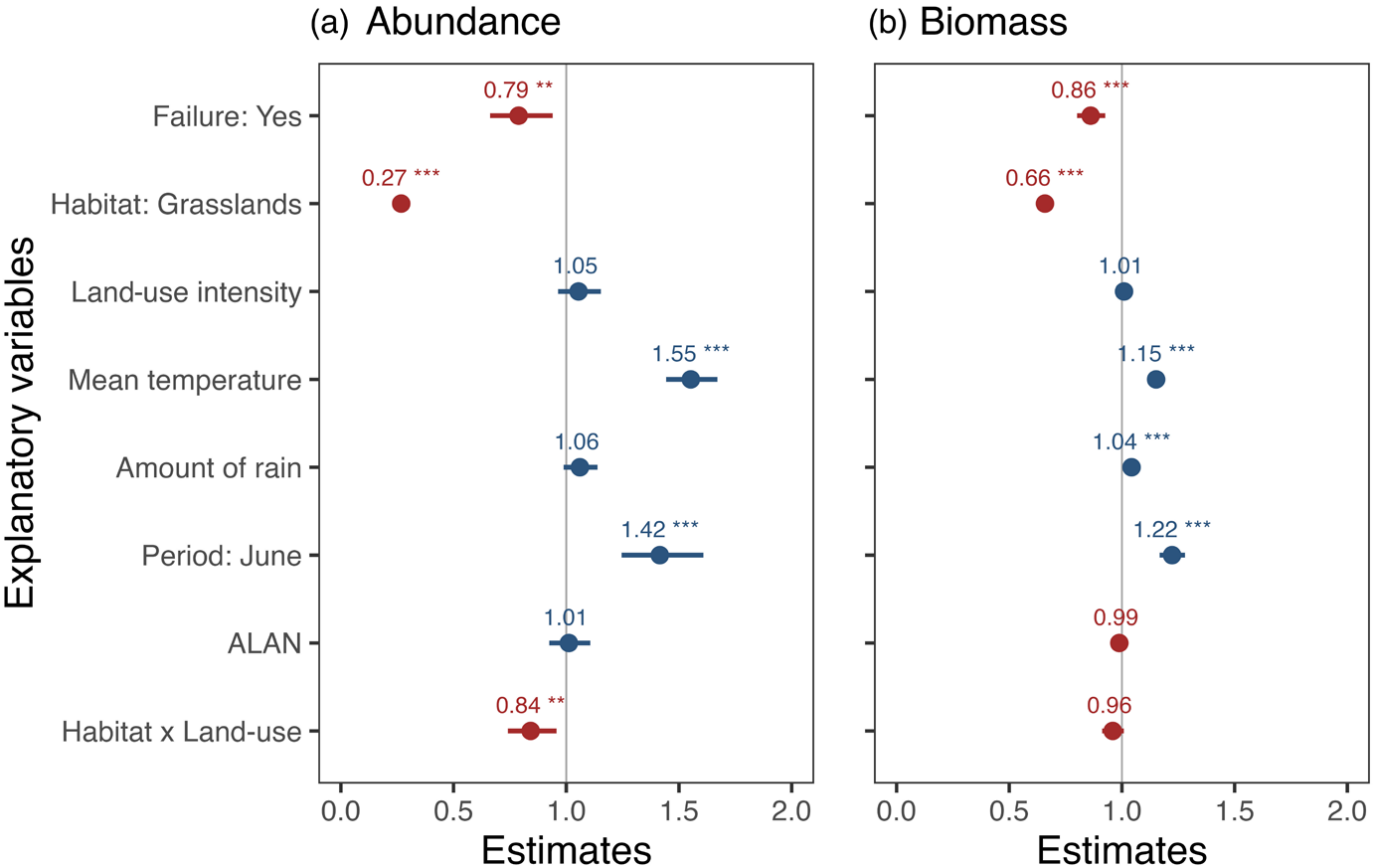
Effect of the selected explanatory variables affecting: (a) abundance and (b) biomass, in forest and grassland plots across Germany. Plots show standardized effect sizes along with 95% confidence interval based on GLMMs using a negative binomial error. Positive estimates are shown in blue, and negative estimates in red. Statistical significance is indicated by asterisks: *p < 0.05, **p < 0.01, ***p < 0.001. GLMM, generalized linear mixed‐effect model.

When the abundances from all 584 trap‐nights were analysed, mean temperature during the sampling night had a strong positive effect on moth abundances (*z* = 11.82, *p* < 0.001), whereas total rainfall during the sampling night and ALAN had no significant influence (Figure 1a). Additionally, the interaction between LUI and habitat type was significant (*χ* ^2^ = 6.96, df = 1, *p* = 0.008). Moth abundances were consistently higher in forest plots than in grasslands (*z* = −19.60, *p* < 0.001), and while abundance increased with LUI in forests, it declined with increasing LUI in grasslands (*z* = −2.64, *p* = 0.008; Figure 1a; Figure S7). Traps that showed signs of failure captured lower moth abundances (*z* = −2.65, *p* = 0.008) (Figure 1a). Sampling period also had a marked effect, with significantly more moths captured in June than in July/August (*z* = 5.33, *p* < 0.001; Figure 1a).

Average biomass of moths was 15.3 ± 15.3 g per trap‐night in forest plots and 4.4 ± 4.6 g in grassland plots. Although moth abundance and biomass were strongly correlated (Spearman: *ρ* = 0.92; *p* < 0.001), the interaction between habitat and land use became non‐significant, and just habitat remained significant with higher biomass in forests than in grasslands (*z* = −16.25; *p* < 0.001) (Figure 1b). Moth biomass increased with higher amounts of rain (*z* = 3.31; *p* < 0.001) and increasing mean temperature (*z* = 11.05; *p* < 0.001) during the sampling night, and was higher in June than in July/August (*z* = 8.58; *p* < 0.001) (Figure 1b).

### 3.3 Sample coverage and alpha diversity (Q2)

Sample coverage ranged from 37% to 99%, with an average of 82% in the 291 plots (Figure S8). Four variables significantly affected coverage negatively: mean winter temperature (*z* = −2.81; *p* = 0.005), amount of rain during the growing season (*z* = −2.54; *p* = 0.011) and LUI (*z* = −2.76; *p* = 0.006). Coverage was lower in grassland than in forests (*z* = −12.56; *p* < 0.001). An increasing percentage of grasslands in the surroundings of a plot significantly increased coverage (*z* = 4.13; *p* < 0.001) (Figure S9).

**FIGURE 2:**
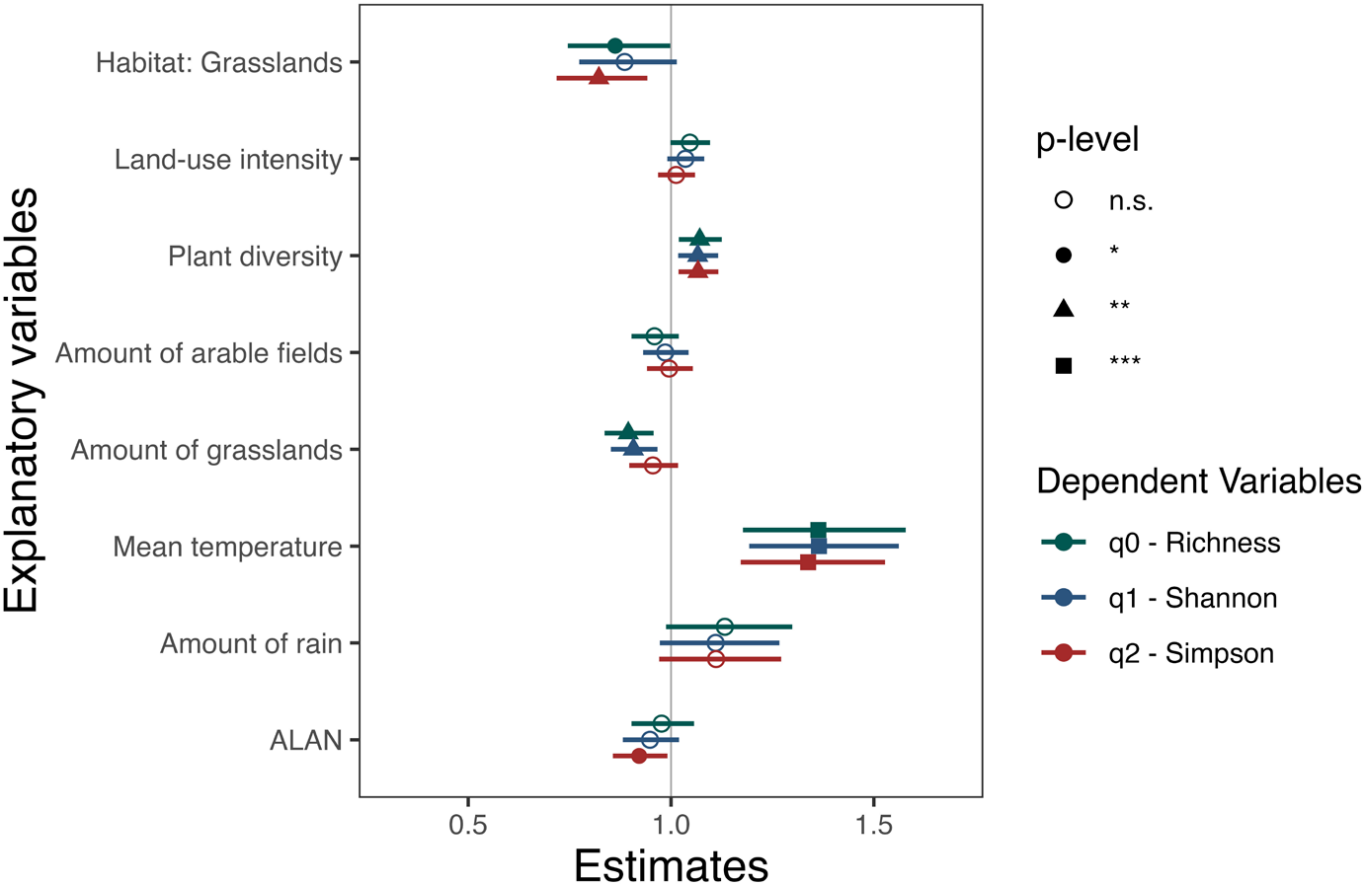
Results of the alpha‐diversity analysis for coverage at 90%. Effect of the selected explanatory variables affecting the diversity of moths in forest and grassland plots across Germany. Plots show standardized effect sizes along with 95% confidence interval based on a GLMM with a negative binomial error structure. Statistical significance is indicated by asterisks and symbols: *p < 0.05, **p < 0.01, ***p < 0.001. GLMM, generalized linear mixed‐effect model.

Moth diversity was higher in forests than in grasslands, for species richness (*q* = 0) and Simpson diversity (*q* = 2), especially at 90% coverage, but not for Shannon diversity. Plant diversity and mean winter temperature significantly increased moth diversity across all Hill numbers (species richness, Shannon and Simpson diversity) (Figure 2; Table S5). In contrast, an increasing percentage of grasslands in the surroundings significantly reduced species richness (*q* = 0) and Shannon diversity (*q* = 1), but had no significant effect on Simpson diversity (*q* = 2). Increased levels of ALAN significantly reduced diversity (*q* = 2) by promoting the dominance of a few species, but had no effect on species richness or Shannon diversity. LUI and the percentage of arable fields in the surroundings had no significant relationship with any diversity level. Generally, conducting the analyses at a higher level of coverage (90% vs. 70%) resulted in more variables becoming statistically significant (Figure 2; Table S5). A sensitivity analysis, including all plots, regardless of moth abundance, yielded consistent ecological patterns, reinforcing the robustness of our results while highlighting minor variability for variables with smaller or non‐significant effects (Table S6; Supporting Information for additional discussion).

### 3.4 Beta diversity (Q3)

Moth community dissimilarity increased with increasing geographic distance (MRM: *p* = 0.001), habitat type (*p* = 0.001), increasing LUI (*p* = 0.025) and an increasing amount of grasslands in the surrounding (*p* = 0.003) (Table 1). In contrast, increasing dissimilarity in mean winter temperature (*p* = 0.001), amount of rain during the growing season (*p* = 0.001) and ALAN (*p* = 0.001) reduced moth community dissimilarity, after controlling for the effects of the other variables. All these results were consistent along the Hill series, with stronger effects across diversity orders (i.e. *q* = 0, *q* = 1 and *q* = 2) (Table 1). This suggests that the variables examined exerted greater influence on the turnover of rare species than only of common species. Plant diversity (*p* = 0.073) and the amount of surrounding arable land (*p* = 0.088) showed weak and only marginally significant effects. In addition, beta diversity was high across all plots (Figure S10), driven almost entirely by turnover (>98%), indicating strong species replacement across plots with minimal nestedness.

**TABLE 1:** Multiple predictors of moth dissimilarity.

Variables	Sørensen index (*q* = 0)	Horn index (*q* = 1)	Morisita–Horn index (*q* = 2)
Geographic distance	**0.062**; *p* **= 0.001**	**0.057**; *p* **= 0.001**	**0.050**; *p* < **0.001**
Habitat: Grassland	**0.149**; *p* = **0.001**	**0.131**; *p* = **0.001**	**0.109**; ***p*** < **0.001**
Land‐use intensity	**0.006**; *p* = **0.025**	**0.008**; *p* = **0.004**	**0.008**; *p* < **0.001**
Plant diversity	−0.005; *p* = 0.073	−0.003; *p* = 0.180	−0.002; *p* = 0.410
Amount of arable fields	0.005; *p* = 0.088	0.003; *p* = 0.154	0.002; *p* = 0.360
Amount of grasslands	**0.008**; *p* = **0.003**	**0.007**; *p* = **0.004**	**0.006**; *p* < **0.005**
Mean temperature	**−0.015**; *p* = **0.001**	**−0.014**; *p* = **0.001**	**−0.012**; *p* < **0.001**
Amount of rain	**−0.025**; *p* = **0.001**	**−0.022**; *p* = **0.001**	**−0.017**; *p* < **0.001**
ALAN	**−0.018**; *p* = **0.001**	**−0.014**; *p* = **0.001**	**−0.010**; *p* < **0.001**
*R* ^2^	0.560	0.544	0.509

*Note*: Results of the beta‐diversity analysis based on MRM for the moth species composition calculated from Hill numbers 0, 1 and 2. Bold values (*p* < 0.05) indicate statistically significant results. Significance values were calculated by permutation using MRM, which tests for the significance of each predictor variable after controlling for the effect of the other variables in the model. *N* pairs of plots = 40,470.

Abbreviations: ALAN, artificial light at night; MRM, multiple regression on distance matrices.

**FIGURE 3:**
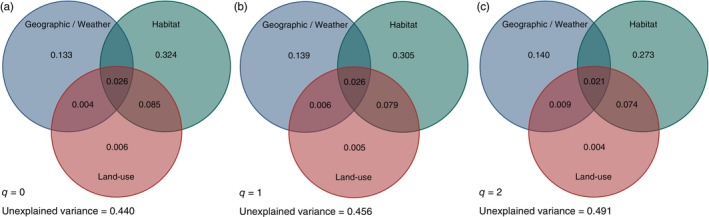
Venn diagram showing the relative contribution (proportion) of our main predictor variables to explain the variation in moth dissimilarity, calculated using variance partitioning. Variables were grouped in three categories: (1) *Geographic/weather*, representing the geographic distance and the seasonal weather condition; (2) *Habitat*, which covers the habitat type and plant diversity; and (3) Land use, representing plot‐ and landscape‐scale variables together with ALAN. Overlapping areas represent variance that is jointly explained by one or more group of predictor variables. In (a), the relative contributions of the three groups of variables on moth dissimilarity calculated based on Hill number 0 (Sørensen index); in (b), the results for Hill number 1 (Horn index); and in (c), the results for Hill number 2 (Morisita–Horn index). ALAN, artificial light at night.

Variance partitioning revealed that the unique and shared contributions of the predictor categories explained 56% (*q* = 0), 54.5% (*q* = 1) and 50.9% (*q* = 2) of the variance in community dissimilarity, with unexplained variance increasing along the Hill series (Figure 3). Habitat‐related variables (i.e. habitat type and plant diversity) explained on average the largest unique portion of moth beta‐diversity variance (31.1%), followed by geographic/weather variables (13.7%). Land‐use variables explained a smaller fraction of variance (0.5%), but a large proportion of the variance explained by land‐use variables (7.9%) was shared with habitat variables (see the overlapping areas in Figure 3), which suggests that differences in moth dissimilarity between forests and grasslands also reflect finer‐scale variations in LUI at both the plot and landscape levels.

## 4 DISCUSSION

Understanding how moth communities respond to habitat transformation and environmental gradients is crucial for biodiversity conservation. Our study provides a comprehensive assessment across 300 temperate plots, integrating habitat type, LUI at multiple spatial scales, weather conditions and light pollution to evaluate their independent and combined effects on multiple diversity dimensions (alpha diversity and beta diversity at q0, q1, q2). While previous studies mostly have focused on single habitats, we demonstrate that grasslands harbour lower moth abundance, biomass and diversity than forests. As expected for ectothermic organisms, weather conditions, particularly temperature measured both during sampling and over the preceding winter, played a key role in shaping moth communities by increasing abundance and diversity. Notably, we show that LUI at the landscape level, especially the amount of grassland surrounding each plot, had a stronger influence on moth communities than local LUI. This suggests that habitat composition and LUI at larger spatial scales play a key role in shaping moth biodiversity. Moreover, spatial beta diversity, once geographic distance was controlled for, was largely explained by habitat change (i.e. from forest to grassland), with species turnover likely reflecting environmental filtering.

Habitat type was a key driver of moth community structure, with grasslands harbouring lower moth abundance, biomass and diversity than forests. This aligns with previous research indicating that structurally complex habitats, such as forests, support higher species richness and diversity (Merckx, Feber, et al., 2012), which might be explained by greater resource heterogeneity, including larval host plants and microclimatic stability (Dantas de Miranda et al., 2019). Additionally, forests provide better habitat quality, as measured by higher plant diversity, which enhances resource availability and supports a wider range of herbivorous insects (Erdős et al., 2023; Summerville & Crist, 2004). However, this holds true for rare species (q0) and dominant species (q2), while common species (q1) did not show significant differences in diversity between forests and grasslands, suggesting that factors influencing common species are less habitat‐specific. The dominance of generalist moth species across habitats (62.9% of the species occurred in both grasslands and forests) suggests they play a key role in shaping community structure, aligning with findings that generalists exhibit higher abundance and resilience to environmental change (Börschig et al., 2013; Mangels et al., 2017). However, the presence of specialist species, 24.6% found exclusively in forests and 12.5% in grasslands, highlights the importance of habitat heterogeneity in preserving functional diversity and maintaining ecosystem stability (Sperandii et al., 2025). Forests supported higher moth biomass, likely because larger‐bodied species tend to prefer more closed‐canopy environments (La Cava et al., 2024), potentially due to reduced predation pressure compared with open environments.

Land‐use intensity at the plot level did not have a significant impact in moth communities, except for moth abundance. In forests, higher LUI was associated with increased moth abundance, likely due to greater resource availability, structural complexity and disturbance regimes. For example, selective logging and the introduction of tree species with a less dense canopy structure can enhance habitat heterogeneity by increasing light penetration and promoting understorey vegetation and productivity (Fonseca et al., 2005), which might support more moth individuals (Heidrich et al., 2020). In contrast, intensive grassland management, characterized by frequent mowing, high grazing pressure and fertilization, disrupts moth life cycles, reduces host plant diversity and overwintering sites for larvae, and affects structural complexity and nectar resources for adults, ultimately leading to declines in moth abundance with increasing LUI (Mangels et al., 2017; Sanetra et al., 2024).

While many studies have demonstrated the strong negative impacts of intensive agriculture on insects (Outhwaite et al., 2022; Raven & Wagner, 2021; Seibold et al., 2019), our results suggest that landscape‐scale effects on moths were primarily driven by the amount of grassland surrounding the plots rather than the amount of arable fields. The observed negative relationship between increasing grassland cover and species richness (q0) and Shannon diversity (q1) indicates that land‐use intensification at the landscape reduces the presence of both rare and common species, likely by limiting habitat connectivity and resource availability (Fuentes‐Montemayor et al., 2017; Newbold et al., 2018). However, dominant species appeared less affected, responding similarly in both habitats to amount of grasslands in the surrounding.

Weather conditions, particularly temperature, strongly influenced moth communities. Warmer nights increased moth abundance and biomass, consistent with metabolic theory, which predicts higher ectotherm activity under elevated temperatures (Prather et al., 2018). This pattern is well documented in temperate regions, where rising temperatures often correlate with higher moth abundances (Froidevaux et al., 2019; Thomsen et al., 2016). Additionally, moth diversity responded positively to warmer mean winter temperatures, likely reflecting improved overwintering survival and enhanced population growth through accelerated metabolism, faster development and higher reproductive success (Zhang et al., 2015). Rainfall during the sampling night also positively influenced moth biomass, likely by creating humid conditions that benefit larger‐bodied moths. This supports findings that rainfall helps moderate ambient temperatures and reduces desiccation risk (Contreras et al., 2013). However, the exact mechanisms behind this relationship remain unclear, highlighting the need for further research on the role of humidity in nocturnal insect activity. We observed a seasonal shift in moth abundance and biomass, with a decline from early (June) to late summer (August), which may reflect short‐term changes in nectar availability as plants senesce (Balfour et al., 2018). This pattern, with higher moth richness and abundance in early‐to‐mid summer, has been linked to phenological peaks in floral resources (Jonason et al., 2014).

In contrast to studies reporting negative effects of ALAN on moth abundance and diversity (e.g. Knop et al., 2017), our results show that only Simpson diversity (*q* = 2), which reflects community dominance, was significantly affected. This indicates a shift in the relative abundance structure rather than a loss of species, with a few species becoming increasingly dominant under elevated ALAN. The three study regions in Central Europe have long been exposed to high levels of light pollution, which may have already led to the local extinction of the most ALAN‐sensitive species (Kalinkat et al., 2021). Consequently, further increases in ALAN in these regions appear to primarily alter species dominance rather than reduce species richness. Notably, the range of ALAN values across our sites was relatively narrow (0.21–0.43, with a mean of 0.27; Figure S11), which may have limited our ability to detect stronger community‐level responses. These findings highlight the need for further research on how ALAN shapes nocturnal biodiversity across broader ecological and spatial gradients, particularly by incorporating species‐level traits such as dispersal capacity, phototactic behaviour and habitat specialization that may mediate differential responses to light pollution.

Moth community composition was shaped by geographic distance, habitat type, LUI at the plot scale and amount of grassland around plots, indicating that spatial and environmental gradients interact to shape moth beta diversity. These findings reinforce well‐established theories on dispersal limitation and habitat filtering (Morlon et al., 2008; Soininen et al., 2007). The observed patterns of high spatial beta diversity suggest that moth communities are shaped by neutral processes, such as dispersal limitation, but also by deterministic processes like environmental filtering (Bae et al., 2021; Cadotte & Tucker, 2017; Chase & Myers, 2011). For instance, the strong distance–decay relationship observed, where community similarity declined with geographic distance, suggests that despite their generally high mobility, moth dispersal may still be constrained by landscape features or species‐specific traits (Püls et al., 2025) across large spatial scales (Beck & Khen, 2007). Habitat type had a strong influence on species turnover. Thus, forests and grasslands provided contrasting environmental conditions (i.e. habitat filtering) harbouring distinct moth assemblages, thereby contributing significantly to landscape‐level biodiversity. Furthermore, high spatial beta diversity of moths was associated with differences in LUI at the plot level, as well as the landscape level, quantified as the amount of surrounding grassland (i.e. spatial configuration), a pattern widely documented for several groups of insects (Gossner et al., 2016; Seibold et al., 2023). Habitat type alone explained the largest unique portion of beta‐diversity variance, and changes in community composition may often reflect broader habitat transitions rather than direct management effects. However, our results also suggest that changes in beta diversity of moth communities is better explained by the combined effect of habitat change and intensification. This effect is slightly stronger for rare species and diminishes for common and dominant species, showing that shifts in community composition may be subtle unless analysed across diversity orders. Importantly, beta‐diversity partitioning based on abundance data revealed that community dissimilarity was overwhelmingly driven by species turnover rather than nestedness. This means that differences in moth communities between plots are largely due to the replacement of species, rather than plots representing subsets with fewer individuals. Such patterns reinforce the role of environmental filtering, and spatial gradients as key mechanisms structuring moth assemblages across the habitats (Soininen et al., 2017).

Our results suggest that dissimilarity in weather conditions may contribute to community homogenization. Specifically, our MRM analysis showed that increased dissimilarity in temperature, rainfall and ALAN was significantly associated with reduced beta diversity, indicating that greater environmental variation among plots may favour overlapping species pools. While environmental heterogeneity is generally expected to increase beta diversity by promoting species turnover (Heino & Grönroos, 2017), it can occasionally reduce it by filtering communities towards species tolerant of a wide range of conditions, leading to greater compositional similarity (Valtonen et al., 2017). However, we acknowledge the limitations of this interpretation. Our data span only 1 year, and seasonal weather was characterized using mean temperature and total precipitation, potentially overlooking important aspects, such as extremes or variability. Furthermore, the relationship between climate and beta diversity may change over time, as shown in long‐term studies on other taxa (Seibold et al., 2023). Future research should address these limitations through multi‐year monitoring and finer‐resolution climate metrics, including anomalies and extremes (Welti et al., 2022), to better disentangle the relative importance of deterministic versus stochastic processes in shaping moth communities.

Our study revealed significant variation in sample coverage across habitat types, land‐use intensities and seasonal conditions, highlighting the importance of standardized sampling approaches and coverage‐based diversity estimation (Chao & Jost, 2012). Notably, coverage was higher in plots surrounded by a greater amount of the same habitat type, likely because homogeneous landscapes facilitate detection of habitat‐specialist species, resulting in more complete samples. In contrast, land‐use intensification, weather variability and heterogeneous surroundings tended to reduce coverage, reflecting the presence of more rare or transient species and increased sampling uncertainty. These patterns caution against simple cross‐habitat diversity comparisons without accounting for uneven sample completeness and underscore the value of rarefaction and extrapolation methods for robust biodiversity assessment. A limitation of our study is the relatively short sampling window, which may not fully capture interannual variation in moth diversity. However, the extensive spatial coverage, 300 plots representing the full gradient of land use and habitat structure, provides a robust space‐for‐time substitution to detect general biodiversity responses (Blüthgen et al., 2022). Nevertheless, future research should assess whether these patterns hold across multiple years and how species‐specific traits mediate moth responses to land‐use change.

## 5 CONCLUSION

Habitat type is the primary determinant of moth community composition, with forests supporting greater abundance, diversity and distinct species communities compared to grasslands. Temperature (*short‐term* and *seasonal*) emerged as a key factor positively influencing moth activity, biomass and alpha‐ and beta diversity. While land‐use intensification interacts with habitat type to affect moth abundance, positively in forests and negatively in grasslands, the negative effects of intensification on moth diversity were more pronounced at the landscape level than at the plot level, particularly when adjacent grasslands dominate surrounding areas. Spatial beta diversity was affected mainly by spatial distance among plots and habitat type, suggesting both dispersal limitation and environmental filtering. Overall, our results highlight the complex interplay of habitat characteristics, LUI and weather in shaping moth communities. Importantly, we found that compositional turnover responds more consistently than local richness to these environmental drivers, reinforcing the idea that biodiversity change is often masked when only alpha diversity (in particular species richness) is considered. Conservation strategies for moths should therefore focus on maintaining habitat heterogeneity, promoting plant diversity and mitigating the negative effects of land‐use intensification, particularly at the landscape level where species diversity and identity are more vulnerable to management practices. The positive effects of spatial distance on beta diversity further suggest that conservation efforts should encompass larger areas to capture wider environmental variation and preserve vulnerable, habitat‐specific species before compositional homogenization occurs.

## AUTHOR CONTRIBUTIONS

Sebastian Seibold, Jörg Müller, Lea Heidrich and Wolfgang Weisser conceived the ideas and designed the methodology. Sebastian Seibold, Lea Heidrich, Hermann Hacker, Markus Fisher and Wolfgang Weisser collected the data. Rafael Achury, Michael Staab, Jörg Müller, Marcel Püls, Carlos Roberto Fonseca, Nico Blüthgen and Wolfgang Weisser planned the analyses. Rafael Achury analysed the data and led the writing of the manuscript. All authors contributed critically to the drafts and gave final approval for publication.

## CONFLICT OF INTEREST STATEMENT

The authors declare no conflict of interest.

## Supporting information


**Table S1.** Values of land use intensity measurement and their associated scaled‐value (*z*‐transformation) in grassland and forests for the three regions sampled: Swabian Alb (yellow shade), Hainich‐Dün (blue), and Schorfheide‐Chorin (red).
**Table S2.** Results of generalized linear mixed‐effects models comparing moth abundances across predictor variables under different trap inclusion thresholds.
**Table S3.** Results of generalized linear mixed‐effects models comparing moth biomass across predictor variables under different trap inclusion thresholds.
**Table S4.** Abundance of moth species in grassland and forests for the three regions sampled: Swabian Alb (yellow shade), Hainich‐Dün (blue), and Schorfheide‐Chorin (red).
**Table S5.** Results of the alpha diversity analysis.
**Table S6.** Results of the sensitivity analysis including all plots (without excluding those with low moth abundance).
**Figure S1.** The study was conducted in three regions of Germany: Schorfheide‐Chorin, Hainich‐Dün, and Schwäbische Alb.
**Figure S2.** Portable automated light traps located in a forest plot.
**Figure S3.** Collinearity based on correlation matrix (a) and values of the variance inflation factor (VIF) (b) used to evaluate how moth diversity was affected by the predictor variables associated with habitat, plot‐ and landscape‐scale land‐use intensity and seasonal weather conditions.
**Figure S4.** Number of moth individuals across three regions in Germany (Alb, Hainich, and Schorfheide) for two distinct habitats: grassland and forest.
**Figure S5.** Number of moth species across three regions in Germany (Alb, Hainich, and Schorfheide) for two distinct habitats: grassland and forest.
**Figure S6.** Number of species detected in each of the habitats per region (number in parentheses).
**Figure S7.** Effect of the land use intensity and its interaction with habitat on moth abundance captured in 584 traps‐nights.
**Figure S8.** Histograms of sampling coverage for moth diversity assessments in forest and grassland habitats.
**Figure S9.** Standardized model estimates (±95% confidence intervals) for the effects of explanatory variables on sample coverage.
**Figure S10.** Partitioning of abundance‐based multiple‐site dissimilarity.
**Figure S11.** Distribution of artificial light at night (ALAN) intensity across study plots in two habitat types: forest (green) and grassland (orange).

## Data Availability

This work is based on data elaborated by a project of the Biodiversity Exploratories program (DFG Priority Program 1374). The datasets are publicly available in the Biodiversity Exploratories Information System (http://doi.org/10.17616/R32P9Q) under the accession numbers: 26026 (moths), 31855 (land‐use forest), 24247 (vegetation grassland), 28886 (vegetation forest) and 19007 (climate).
