# Supplementary material for: Habitat and land‐use intensity shape moth community structure across temperate forest and grassland
Source: J Anim Ecol. 2025 Sep 9;94(11):2308–21. doi: 10.1111/1365-2656.70132 (PMC12586792; doi:10.1111/1365-2656.70132)
Supplement: Supplementary file 1 — Table S1. Values of land use intensity measurement and their associated scaled‐value (z‐transformation) in grassland and forests for the three regions sampled: Swabian Alb (yellow shade), Hainich‐Dün (blue), and Schorfheide‐Chorin (red). Table S2. Results of generalized linear mixed‐effects models comparing moth abundances across predictor variables under different trap inclusion thresholds. Table S3. Results of generalized linear mixed‐effects models comparing moth biomass across predictor variables under different trap inclusion thresholds. Table S4. Abundance of moth species in grassland and forests for the three regions sampled: Swabian Alb (yellow shade), Hainich‐Dün (blue), and Schorfheide‐Chorin (red). Table S5. Results of the alpha diversity analysis. Table S6. Results of the sensitivity analysis including all plots (without excluding those with low moth abundance). Figure S1. The study was conducted in three regions of Germany: Schorfheide‐Chorin, Hainich‐Dün, and Schwäbische Alb. Figure S2. Portable automated light traps located in a forest plot. Figure S3. Collinearity based on correlation matrix (a) and values of the variance inflation factor (VIF) (b) used to evaluate how moth diversity was affected by the predictor variables associated with habitat, plot‐ and landscape‐scale land‐use intensity and seasonal weather conditions. Figure S4. Number of moth individuals across three regions in Germany (Alb, Hainich, and Schorfheide) for two distinct habitats: grassland and forest. Figure S5. Number of moth species across three regions in Germany (Alb, Hainich, and Schorfheide) for two distinct habitats: grassland and forest. Figure S6. Number of species detected in each of the habitats per region (number in parentheses). Figure S7. Effect of the land use intensity and its interaction with habitat on moth abundance captured in 584 traps‐nights. Figure S8. Histograms of sampling coverage for moth diversity assessments in forest and grassland habitats. Figure S9 [file JANE-94-2308-s001.docx]

**Supporting Information**

**Habitat and land-use intensity shape moth community structure across temperate forest and grassland**

Rafael Achury^1^; Michael Staab^2,3^ Sebastian Seibold^4,1^; Jörg Müller^5,6^; Lea Heidrich^7^; Marcel Püls^5,6^; Hermann Hacker^5,6^; Carlos Roberto Fonseca^8^; Markus Fischer^9^; Nico Blüthgen^2^; Wolfgang Weisser^1^

**Table S1.** Values of land use intensity measurement and their associated scaled-value (z-transformation) in grassland and forests for the three regions sampled: Swabian Alb (yellow shade), Hainich-Dün (blue), and Schorfheide-Chorin (red).

| **Region** | **Grasslands** | | | **Forests** | | |
| --- | --- | --- | --- | --- | --- | --- |
|  | **Plot ID** | **LUI index** | **Scaled values** | **Plot ID** | **ForMIX Index** | **Scaled values** |
| Swabian Alb | AEG01 | 1.68 | 0.134945676 | AEW01 | 2.5642944 | 1.935217635 |
|  | AEG02 | 2.63 | 1.493939229 | AEW02 | 2.0505541 | 1.050445896 |
|  | AEG03 | 1.19 | -0.566008894 | AEW03 | 2.4194203 | 1.685713185 |
|  | AEG04 | 1.91 | 0.463965168 | AEW04 | 1.6010292 | 0.276266949 |
|  | AEG05 | 1.94 | 0.506880753 | AEW05 | 1.4323694 | -0.014201614 |
|  | AEG06 | 1.78 | 0.277997629 | AEW06 | 1.4939337 | 0.09182544 |
|  | AEG07 | 1.07 | -0.737671238 | AEW07 | 0.3972605 | -1.796882469 |
|  | AEG08 | 1.16 | -0.60892448 | AEW08 | 1.0658723 | -0.645388579 |
|  | AEG09 | 1.13 | -0.651840066 | AEW09 | 1.1501432 | -0.500256047 |
|  | AEG10 | 0.84 | -1.06669073 | AEW10 | 2.4211139 | 1.688629873 |
|  | AEG11 | 2.01 | 0.607017121 | AEW11 | 2.362726 | 1.588073292 |
|  | AEG12 | 1.5 | -0.12254784 | AEW12 | 2.0660353 | 1.077107896 |
|  | AEG13 | 1.84 | 0.3638288 | AEW13 | 2.1541017 | 1.228777196 |
|  | AEG14 | 2.3 | 1.021867784 | AEW14 | 2.0999804 | 1.13556867 |
|  | AEG15 | 2.79 | 1.722822354 | AEW15 | 1.4043187 | -0.06251089 |
|  | AEG16 | 2.01 | 0.607017121 | AEW16 | 1.3420609 | -0.1697322 |
|  | AEG17 | 1.68 | 0.134945676 | AEW17 | 1.6185481 | 0.306438368 |
|  | AEG18 | 2.27 | 0.978952198 | AEW18 | 1.1250555 | -0.54346234 |
|  | AEG19 | 1.96 | 0.535491144 | AEW19 | 1.3469237 | -0.161357488 |
|  | AEG20 | 1.36 | -0.322820574 | AEW20 | 1.3631824 | -0.133356572 |
|  | AEG21 | 3.14 | 2.223504189 | AEW21 | 0.8433707 | -1.028584462 |
|  | AEG22 | 0.84 | -1.06669073 | AEW22 | 1.3322483 | -0.186631659 |
|  | AEG23 | 1.19 | -0.566008894 | AEW23 | 1.4457874 | 0.008907193 |
|  | AEG24 | 2.37 | 1.122004151 | AEW24 | 1.2153929 | -0.387881975 |
|  | AEG25 | 0.75 | -1.195437487 | AEW25 | 1.5781058 | 0.236788 |
|  | AEG26 | 2.17 | 0.835900245 | AEW26 | 1.5008785 | 0.103785969 |
|  | AEG27 | 1.29 | -0.422956941 | AEW27 | 1.4104564 | -0.051940483 |
|  | AEG28 | 0.78 | -1.152521901 | AEW28 | 1.567334 | 0.218236592 |
|  | AEG29 | 1.73 | 0.206471652 | AEW29 | 1.9835668 | 0.935079265 |
|  | AEG30 | 1.29 | -0.422956941 | AEW30 | 1.4889635 | 0.08326574 |
|  | AEG31 | 1.48 | -0.15115823 | AEW31 | 2.7378681 | 2.234148999 |
|  | AEG32 | 0.49 | -1.567372565 | AEW32 | 2.6455033 | 2.075076765 |
|  | AEG33 | 0.8 | -1.123911511 | AEW33 | 2.3563238 | 1.577047271 |
|  | AEG34 | 1.2 | -0.551703699 | AEW34 | 2.5930445 | 1.984731498 |
|  | AEG35 | 2.27 | 0.978952198 | AEW35 | 0.9886685 | -0.778350262 |
|  | AEG36 | 2.27 | 0.978952198 | AEW36 | 0.972775 | -0.805722363 |
|  | AEG37 | 1.98 | 0.564101535 | AEW37 | 1.2666442 | -0.299616111 |
|  | AEG38 | 1.19 | -0.566008894 | AEW38 | 1.7473554 | 0.528272343 |
|  | AEG39 | 1.67 | 0.12064048 | AEW39 | 1.6939231 | 0.436250294 |
|  | AEG40 | 2.05 | 0.664237902 | AEW40 | 1.4213173 | -0.03323559 |
|  | AEG41 | 1.9 | 0.449659972 | AEW41 | 1.3068797 | -0.230321968 |
|  | AEG42 | 1.84 | 0.3638288 | AEW42 | 1.2754781 | -0.284402202 |
|  | AEG43 | 2 | 0.592711925 | AEW43 | 1.2265019 | -0.368749837 |
|  | AEG44 | 1.73 | 0.206471652 | AEW44 | 0.5630984 | -1.511273743 |
|  | AEG45 | 1.56 | -0.036716668 | AEW45 | 1.8535333 | 0.711133517 |
|  | AEG46 | 2.2 | 0.878815831 | AEW46 | 1.7664155 | 0.561097965 |
|  | AEG47 | 1.08 | -0.723366042 | AEW47 | 1.9190739 | 0.824008637 |
|  | AEG48 | 0.83 | -1.080995925 | AEW48 | 1.5532072 | 0.193907216 |
|  | AEG49 | 0.95 | -0.909333581 | AEW49 | 0.9149356 | -0.905334203 |
|  | AEG50 | 1.55 | -0.051021863 | AEW50 | 0.8174817 | -1.073170935 |
| Hainich-Dün | HEG01 | 2.8 | 1.737127549 | HEW01 | 1.8324224 | 0.674776105 |
|  | HEG02 | 2.61 | 1.465328839 | HEW03 | 2.0908788 | 1.11989374 |
|  | HEG03 | 2.61 | 1.465328839 | HEW04 | 1.7799658 | 0.58443441 |
|  | HEG04 | 1.62 | 0.049114504 | HEW05 | 1.3341055 | -0.18343325 |
|  | HEG05 | 2.26 | 0.964647003 | HEW06 | 1.3425409 | -0.168905546 |
|  | HEG06 | 2.21 | 0.893121027 | HEW07 | 1.0226789 | -0.719776969 |
|  | HEG07 | 3.47 | 2.695575634 | HEW08 | 1.03055 | -0.706221231 |
|  | HEG08 | 1.54 | -0.065327059 | HEW09 | 1.1359437 | -0.524710499 |
|  | HEG09 | 0.83 | -1.080995925 | HEW10 | 0.9736457 | -0.804222818 |
|  | HEG10 | 1.52 | -0.093937449 | HEW11 | 0.9225654 | -0.892194099 |
|  | HEG11 | 1.83 | 0.349523605 | HEW12 | 0.6062711 | -1.436921052 |
|  | HEG12 | 3.18 | 2.280724971 | HEW13 | 2.1179409 | 1.166500498 |
|  | HEG13 | 1.57 | -0.022411473 | HEW14 | 1.4204192 | -0.034782341 |
|  | HEG14 | 1.63 | 0.063419699 | HEW15 | 1.1324867 | -0.530664284 |
|  | HEG15 | 2 | 0.592711925 | HEW16 | 1.1981907 | -0.417507859 |
|  | HEG16 | 1.12 | -0.666145261 | HEW17 | 1.1790717 | -0.450434929 |
|  | HEG17 | 0.51 | -1.538762174 | HEW18 | 1.7458992 | 0.525764395 |
|  | HEG18 | 0.53 | -1.510151784 | HEW19 | 1.2136517 | -0.390880703 |
|  | HEG19 | 0.23 | -1.939307643 | HEW20 | 1.1885703 | -0.434076205 |
|  | HEG20 | 0.83 | -1.080995925 | HEW21 | 1.3333449 | -0.184743038 |
|  | HEG21 | 0.8 | -1.123911511 | HEW22 | 1.1310848 | -0.533078589 |
|  | HEG22 | 1.21 | -0.537398503 | HEW23 | 0.9900849 | -0.775910873 |
|  | HEG23 | 1.16 | -0.60892448 | HEW24 | 0.8344891 | -1.043880525 |
|  | HEG24 | 1.28 | -0.437262136 | HEW25 | 1.3429811 | -0.168147467 |
|  | HEG25 | 1.27 | -0.451567332 | HEW26 | 0.9283154 | -0.882291253 |
|  | HEG26 | 2.17 | 0.835900245 | HEW27 | 1.1536 | -0.494302601 |
|  | HEG27 | 1.98 | 0.564101535 | HEW28 | 1.2777586 | -0.280474739 |
|  | HEG28 | 1.86 | 0.392439191 | HEW29 | 1.3420643 | -0.16972637 |
|  | HEG29 | 1.73 | 0.206471652 | HEW30 | 1.1665822 | -0.471944383 |
|  | HEG30 | 2.09 | 0.721458683 | HEW31 | 1.205127 | -0.405562012 |
|  | HEG31 | 1.83 | 0.349523605 | HEW32 | 0.9819367 | -0.789943932 |
|  | HEG32 | 1.92 | 0.478270363 | HEW33 | 1.366537 | -0.127579085 |
|  | HEG33 | 1.62 | 0.049114504 | HEW34 | 0.8100654 | -1.085943278 |
|  | HEG34 | 2.21 | 0.893121027 | HEW35 | 1.0075615 | -0.745812458 |
|  | HEG35 | 2.64 | 1.508244424 | HEW36 | 1.0495736 | -0.67345852 |
|  | HEG36 | 1.65 | 0.09203009 | HEW37 | 0.3929467 | -1.804311802 |
|  | HEG37 | 1.21 | -0.537398503 | HEW38 | 0.7669483 | -1.160200233 |
|  | HEG38 | 1.57 | -0.022411473 | HEW39 | 0.6385943 | -1.381253536 |
|  | HEG39 | 1.4 | -0.265599793 | HEW40 | 1.0118916 | -0.73835499 |
|  | HEG40 | 1.62 | 0.049114504 | HEW41 | 0.8859565 | -0.955242538 |
|  | HEG41 | 0.83 | -1.080995925 | HEW42 | 0.7788085 | -1.13977448 |
|  | HEG42 | 0.19 | -1.996528424 | HEW43 | 1.6445666 | 0.351247818 |
|  | HEG43 | 0.36 | -1.753340104 | HEW44 | 1.2878327 | -0.263124951 |
|  | HEG44 | 0.92 | -0.952249167 | HEW45 | 1.7411537 | 0.517591601 |
|  | HEG45 | 0.95 | -0.909333581 | HEW46 | 1.2516052 | -0.325516578 |
|  | HEG46 | 0.77 | -1.166827097 | HEW47 | 1.1668208 | -0.471533614 |
|  | HEG47 | 1.94 | 0.506880753 | HEW48 | 0.8874874 | -0.95260591 |
|  | HEG48 | 1.72 | 0.192166457 | HEW49 | 1.2296477 | -0.363332116 |
|  | HEG49 | 1.52 | -0.093937449 | HEW50 | 0.9882287 | -0.779107657 |
|  | HEG50 | 1.5 | -0.12254784 | HEW51 | NA | NA |
| Schorfheide-Chorin | SEG01 | 1.54 | -0.065327059 | SEW01 | 2.5675608 | 1.940843034 |
|  | SEG02 | 0.99 | -0.8521128 | SEW02 | 2.2978605 | 1.476360882 |
|  | SEG03 | 1.06 | -0.751976433 | SEW03 | 2.3669821 | 1.595403296 |
|  | SEG04 | 1.54 | -0.065327059 | SEW04 | 1.7044292 | 0.454344086 |
|  | SEG05 | 1.09 | -0.709060847 | SEW05 | 0.7070909 | -1.263287682 |
|  | SEG06 | 1.99 | 0.57840673 | SEW06 | 1.2197588 | -0.380362869 |
|  | SEG07 | 1.12 | -0.666145261 | SEW07 | 0.6882176 | -1.295791521 |
|  | SEG08 | 1.74 | 0.220776847 | SEW08 | 0.7079229 | -1.261854764 |
|  | SEG09 | 1.96 | 0.535491144 | SEW09 | 1.027141 | -0.71209238 |
|  | SEG10 | 1.09 | -0.709060847 | SEW10 | 2.6665702 | 2.111358534 |
|  | SEG11 | 1.1 | -0.694755652 | SEW11 | 2.6667882 | 2.111734041 |
|  | SEG12 | 1.54 | -0.065327059 | SEW12 | 2.5626013 | 1.932301648 |
|  | SEG13 | 3.36 | 2.538218486 | SEW13 | 2.2561512 | 1.40452856 |
|  | SEG14 | 1.59 | 0.006198918 | SEW14 | 2.4837967 | 1.796583125 |
|  | SEG15 | 1.54 | -0.065327059 | SEW15 | 2.4375823 | 1.716992005 |
|  | SEG16 | 1.09 | -0.709060847 | SEW16 | 2.3546249 | 1.574121434 |
|  | SEG17 | 1.82 | 0.33521841 | SEW17 | 2.1548484 | 1.230063285 |
|  | SEG18 | 1.54 | -0.065327059 | SEW18 | 2.4236523 | 1.693001516 |
|  | SEG19 | 1.42 | -0.236989402 | SEW19 | 2.5615588 | 1.930506359 |
|  | SEG20 | 1.69 | 0.149250871 | SEW20 | 2.5602938 | 1.928327659 |
|  | SEG21 | 1.67 | 0.12064048 | SEW21 | 2.2800552 | 1.445696368 |
|  | SEG22 | 0.95 | -0.909333581 | SEW22 | 1.6070028 | 0.286554824 |
|  | SEG23 | 1.54 | -0.065327059 | SEW23 | 1.4545573 | 0.024010764 |
|  | SEG24 | 1.54 | -0.065327059 | SEW24 | 1.123588 | -0.54598969 |
|  | SEG25 | 1.54 | -0.065327059 | SEW25 | 1.5261542 | 0.147316117 |
|  | SEG26 | 1.54 | -0.065327059 | SEW26 | 0.3155853 | -1.937544695 |
|  | SEG27 | 1.17 | -0.594619285 | SEW27 | 0.7843267 | -1.130270859 |
|  | SEG28 | 1.09 | -0.709060847 | SEW28 | 1.4136964 | -0.04636045 |
|  | SEG29 | 1.54 | -0.065327059 | SEW29 | 1.4736193 | 0.056839626 |
|  | SEG30 | 1.13 | -0.651840066 | SEW30 | 1.6857442 | 0.42216457 |
|  | SEG31 | 1.13 | -0.651840066 | SEW31 | 1.6471367 | 0.355674099 |
|  | SEG32 | 1.13 | -0.651840066 | SEW32 | 2.1505939 | 1.222736019 |
|  | SEG33 | 1.29 | -0.422956941 | SEW33 | 1.9173893 | 0.82110739 |
|  | SEG34 | 0.97 | -0.880723191 | SEW34 | 1.7241017 | 0.488224429 |
|  | SEG35 | 3.95 | 3.382225009 | SEW35 | 1.2954283 | -0.250043601 |
|  | SEG36 | 1.14 | -0.637534871 | SEW36 | 1.3454347 | -0.163921798 |
|  | SEG37 | 1.31 | -0.39434655 | SEW37 | 1.2125489 | -0.392779828 |
|  | SEG38 | 2.25 | 0.950341808 | SEW38 | 1.2859159 | -0.266426151 |
|  | SEG39 | 0.73 | -1.224047878 | SEW39 | 1.0403793 | -0.689293038 |
|  | SEG40 | 0 | -2.268327135 | SEW40 | 1.1389664 | -0.519504832 |
|  | SEG41 | 1.31 | -0.39434655 | SEW41 | 0.9071523 | -0.918738653 |
|  | SEG42 | 4.03 | 3.496666571 | SEW42 | 0.5674709 | -1.503743414 |
|  | SEG43 | 3.89 | 3.296393837 | SEW43 | 0.5440217 | -1.544128034 |
|  | SEG44 | 1.26 | -0.465872527 | SEW44 | 1.0964012 | -0.592811327 |
|  | SEG45 | 0.83 | -1.080995925 | SEW45 | 0.9374533 | -0.866553879 |
|  | SEG46 | 1.94 | 0.506880753 | SEW46 | 0.921125 | -0.894674665 |
|  | SEG47 | 1.4 | -0.265599793 | SEW47 | 0.553925 | -1.527072345 |
|  | SEG48 | 2.27 | 0.978952198 | SEW48 | 0.6062066 | -1.437032122 |
|  | SEG49 | 1.16 | -0.60892448 | SEW49 | 0.9813483 | -0.790957206 |
|  | SEG50 | 0.88 | -1.009469948 | SEW50 | 1.372497 | -0.117314793 |

**Table S2.** Results of generalized linear mixed-effects models comparing moth abundances across predictor variables under different trap inclusion thresholds. Analyses retaining only those with at least 5 individuals (550 plots), 10 individuals (536 plots), 15 individuals (510 plots), or 20 individuals (465 plots). The results remained consistent across these analyses, except for the “Trap failure” variable, which became insignificant in all analyses when at least 10 individuals per trap-night were included. Each row represents the model estimate; z-value; and significance level (**p* < 0.05, ***p* < 0.01, ****p* < 0.001) for a given predictor variable. Bold values (*p* < 0.05) indicate statistically significant results. Predictors include whether the trap failed or not, habitat type (grassland vs. other), plot-scale land-use intensity, temperature and rainfall as measurement of short-term climatic conditions, month (June vs. July/August), artificial light at night (ALAN), and an interaction term between habitat and land-use intensity.

| Predictor | Models with different trap inclusion thresholds (Estimate; *z*-value; *P*) | | | | |
| --- | --- | --- | --- | --- | --- |
|  | All traps included | < 5 individuals  excluded | < 10 individuals  excluded | < 15 individuals  excluded | < 20 individuals  excluded |
| Failure: Yes | **-0.237; -2.652 **** | **-0.191; -2.230 *** | -0.122; -1.434 NS | -0.083; -0.988 NS | -0.055; -0.654 NS |
| Habitat: Grassland | **-1.318; -19.604 ***** | **-1.296; -20.106 ***** | **-1.270; -20.052 ***** | **-1.216; -19.759 ***** | **-1.098; -18.220 ***** |
| Land-use intensity | 0.053; 1.155 NS | 0.053; 1.199 NS | 0.054; 1.252 NS | 0.055; 1.333 NS | 0.045; 1.181 NS |
| Temperature | **0.440; 11.816 ***** | **0.429; 12.512 ***** | **0.427; 12.858 ***** | **0.410; 12.475 ***** | **0.390; 11.927 ***** |
| Amount of rain | 0.059; 1.624 NS | 0.048; 1.451 NS | 0.044; 1.385 NS | 0.036; 1.177 NS | 0.018; 0.602 NS |
| Month: June | **0.347; 5.330 ***** | **0.352; 5.920 ***** | **0.369; 6.503 ***** | **0.354; 6.313 ***** | **0.350; 6.173 ***** |
| ALAN | 0.012; 0.252 0.801 | -0.006; -0.124 0.901 | -0.012; -0.267 0.790 | -0.009; -0.211 0.833 | 0.035; 0.837 0.403 |
| Interaction Habitat x Land-use | **-0.172; -2.637 **** | **-0.173; -2.739 **** | **-0.167; -2.667 **** | **-0.179; -2.974 **** | **-0.152; -2.534 *** |

**Table S3.** Results of generalized linear mixed-effects models comparing moth biomass across predictor variables under different trap inclusion thresholds. Correspondingly, when biomass data was analyzed including traps in which we did not have captures, traps with some indication of failure had lower moth biomass. Each row represents the model estimate; z-value; and significance level (**p* < 0.05, ***p* < 0.01, ****p* < 0.001) for a given predictor variable. Bold values (*p* < 0.05) indicate statistically significant results. Predictors include whether the trap failed or not, habitat type (grassland vs. other), plot-scale land-use intensity, temperature and rainfall as measurement of short-term climatic conditions, month (June vs. July/August), artificial light at night (ALAN), and an interaction term between habitat and land-use intensity.

| Predictor | Models with different trap inclusion thresholds (Estimate; *z*-value; *P*) | | | | |
| --- | --- | --- | --- | --- | --- |
|  | All traps included | < 5 individuals  excluded | < 10 individuals  excluded | < 15 individuals  excluded | < 20 individuals  excluded |
| Failure: Yes | **-0.149; -4.036 ***** | **-0.092; -2.672 **** | **-0.081; -2.356 *** | **-0.095; -2.488 *** | **-0.071; -1.966 *** |
| Habitat: Grassland | **-0.418; -16.248 ***** | **-0.394; -16.024 ***** | **-0.380; -15.357 ***** | **-0.364; -14.265 ***** | **-0.333; -13.509 ***** |
| Land-use intensity | 0.009; 0.515 NS | -0.012; -0.986 NS | -0.010; -0.820 NS | -0.012; -0.966 NS | -0.010; -0.789 NS |
| Temperature | **0.141; 11.049 ***** | **0.145; 11.922 ***** | **0.147; 12.068 ***** | **0.147; 11.528 ***** | **0.146; 11.657 ***** |
| Amount of rain | **0.042; 3.311 ***** | **1.056; 3.377 ***** | **1.038; 3.351 ***** | **0.973; 3.128 **** | **0.860; 2.866 **** |
| Month: June | **0.201; 8.579 ***** | **0.192; 8.877 ***** | **0.190; 8.861 ***** | **0.177; 8.055 ***** | **0.178; 7.991 ***** |
| ALAN | -0.012; -0.658 NS | -0.022; -1.282 NS | -0.027; -1.565 NS | -0.028; -1.629 NS | -0.004; -0.234 NS |
| Interaction Habitat x Land-use | -0.042; -1.625 NS | -0.042; -1.722 NS | -0.039; -1.565 NS | -0.041; -1.657 NS | -0.030; -1.204 NS |

**Table S4.** Abundance of moth species in grassland and forests for the three regions sampled: Swabian Alb (yellow shade), Hainich-Dün (blue), and Schorfheide-Chorin (red).

| **Species** | **Swabian Alb** | | **Hainich-Dün** | | **Schorfheide-Chorin** | | **Abundance** |
| --- | --- | --- | --- | --- | --- | --- | --- |
|  | **Grassland** | **Forest** | **Grassland** | **Forest** | **Grassland** | **Forest** |  |
| *Abraxas sylvata* | 3 | 665 | 1 | 57 |  | 8 | 734 |
| *Abrostola asclepiadis* |  | 3 |  |  |  |  | 3 |
| *Abrostola tripartita* |  | 35 |  | 28 |  | 7 | 70 |
| *Abrostola triplasia* |  | 1 |  | 5 |  | 4 | 10 |
| *Acontia trabealis* |  |  |  |  | 10 | 1 | 11 |
| *Acronicta aceris* | 1 | 1 |  |  | 1 |  | 3 |
| *Acronicta auricoma* | 4 | 23 |  |  |  |  | 27 |
| *Acronicta leporina* |  |  | 1 |  |  | 2 | 3 |
| *Acronicta megacephala* | 2 |  | 1 | 2 |  | 6 | 11 |
| *Acronicta psi* |  | 5 | 1 | 2 |  | 2 | 10 |
| *Acronicta rumicis* | 3 | 56 |  | 7 |  | 52 | 118 |
| *Actinotia polyodon* | 3 |  | 4 | 1 |  |  | 8 |
| *Agrotis bigramma* |  |  |  |  | 9 | 4 | 13 |
| *Agrotis clavis* | 117 | 4 | 56 | 1 | 123 | 2 | 303 |
| *Agrotis exclamationis* | 350 | 185 | 237 | 17 | 108 | 30 | 927 |
| *Agrotis ipsilon* | 4 | 5 | 1 | 4 | 7 | 44 | 65 |
| *Agrotis segetum* |  | 2 |  | 1 |  | 39 | 42 |
| *Agrotis vestigialis* |  |  |  |  |  | 17 | 17 |
| *Alcis bastelbergeri* | 1 | 10 |  |  |  |  | 11 |
| *Alcis jubata* |  | 1 |  |  |  |  | 1 |
| *Alcis repandata* | 36 | 1277 | 33 | 253 |  | 115 | 1714 |
| *Amphipoea fucosa* | 40 | 3 | 232 | 7 | 8 |  | 290 |
| *Amphipoea oculea* | 7 |  |  |  | 1 |  | 8 |
| *Amphipyra berbera* | 1 | 1 | 1 | 4 |  | 2 | 9 |
| *Amphipyra perflua* |  | 32 |  |  |  |  | 32 |
| *Amphipyra pyramidea* |  |  | 1 |  |  | 5 | 6 |
| *Amphipyra tragopoginis* | 1 | 3 | 14 | 9 | 3 | 1 | 31 |
| *Anaplectoides prasina* | 52 | 501 |  | 14 | 2 | 2 | 571 |
| *Anarta trifolii* | 1 |  | 4 |  | 84 | 68 | 157 |
| *Angerona prunaria* |  | 25 | 7 | 220 |  | 68 | 320 |
| *Apamea anceps* | 10 |  | 56 | 7 |  |  | 73 |
| *Apamea crenata* | 1 | 1 | 1 | 1 |  |  | 4 |
| *Apamea epomidion* | 1 | 1 |  | 1 |  | 1 | 4 |
| *Apamea monoglypha* | 44 | 38 | 54 | 3 | 9 | 3 | 151 |
| *Apamea ophiogramma* |  |  |  |  | 1 |  | 1 |
| *Apamea remissa* |  |  |  | 1 |  |  | 1 |
| *Apamea scolopacina* | 15 | 114 | 7 | 94 |  |  | 230 |
| *Apamea sordens* | 2 |  | 20 | 1 | 1 |  | 24 |
| *Apamea sublustris* | 14 | 46 | 28 | 2 | 1 |  | 91 |
| *Apamea unanimis* |  |  | 3 |  | 1 |  | 4 |
| *Apeira syringaria* | 1 | 13 |  | 3 |  | 1 | 18 |
| *Aplasta ononaria* |  |  | 15 |  |  |  | 15 |
| *Aplocera plagiata* |  | 3 |  |  | 1 | 15 | 19 |
| *Aplocera praeformata* |  |  |  | 1 |  |  | 1 |
| *Apoda limacodes* | 43 | 716 | 136 | 209 |  | 37 | 1141 |
| *Archanara neurica* |  | 1 |  |  |  |  | 1 |
| *Arctia caja* | 7 | 4 | 10 |  | 15 | 14 | 50 |
| *Arctornis l-nigrum* | 1 | 23 | 12 | 763 |  | 109 | 908 |
| *Arichanna melanaria* | 1 |  |  |  |  |  | 1 |
| *Asthena albulata* | 1 | 5 | 1 | 9 |  | 5 | 21 |
| *Atolmis rubricollis* | 9 | 70 | 107 | 84 |  | 5 | 275 |
| *Atypha pulmonaris* |  | 38 | 1 | 13 |  |  | 52 |
| *Autographa gamma* | 15 | 17 | 12 | 11 | 5 | 16 | 76 |
| *Autographa jota* | 1 | 27 |  |  |  |  | 28 |
| *Autographa pulchrina* | 2 | 23 |  | 14 |  |  | 39 |
| *Axylia putris* | 31 | 17 | 17 | 2 | 11 | 13 | 91 |
| *Biston betularia* | 14 | 108 | 16 | 166 | 1 | 13 | 318 |
| *Brachylomia viminalis* | 2 | 46 | 2 | 1 |  |  | 51 |
| *Bupalus piniaria* |  | 1 | 8 |  | 3 | 60 | 72 |
| *Cabera exanthemata* |  | 7 | 3 |  | 8 | 26 | 44 |
| *Cabera pusaria* | 6 | 42 | 5 | 42 | 31 | 39 | 165 |
| *Calamia tridens* | 3 |  | 6 |  |  |  | 9 |
| *Callierges ramosa* |  |  |  | 4 |  |  | 4 |
| *Callimorpha dominula* |  |  |  | 1 |  | 12 | 13 |
| *Calliteara pudibunda* |  |  |  | 249 |  | 1 | 250 |
| *Callopistria juventina* |  |  |  |  |  | 11 | 11 |
| *Campaea margaritaria* | 176 | 3190 | 146 | 1020 | 9 | 91 | 4632 |
| *Camptogramma bilineata* | 10 | 184 | 11 | 38 | 3 | 419 | 665 |
| *Caradrina morpheus* |  |  | 7 |  | 2 | 3 | 12 |
| *Caradrina selini* |  |  |  |  |  | 1 | 1 |
| *Catarhoe cuculata* | 2 | 23 | 7 | 4 | 1 |  | 37 |
| *Catarhoe rubidata* |  | 3 |  |  |  |  | 3 |
| *Catocala promissa* |  | 1 |  | 2 |  |  | 3 |
| *Catocala sponsa* |  |  | 2 |  |  | 3 | 5 |
| *Celaena leucostigma* |  |  |  |  | 10 |  | 10 |
| *Cepphis advenaria* |  |  |  | 2 |  | 4 | 6 |
| *Ceramica pisi* | 6 |  | 7 |  | 3 | 9 | 25 |
| *Cerapteryx graminis* | 3 | 3 | 1 |  | 11 | 80 | 98 |
| *Charanyca trigrammica* | 16 | 21 | 130 | 19 | 5 | 4 | 195 |
| *Chersotis cuprea* | 1 |  |  |  |  |  | 1 |
| *Chersotis margaritacea* |  | 132 |  |  |  |  | 132 |
| *Chersotis multangula* | 1 | 1 |  |  |  |  | 2 |
| *Chiasmia clathrata* | 194 | 37 | 241 | 9 | 477 | 187 | 1145 |
| *Chloroclystis v-ata* |  | 8 | 1 | 3 | 1 | 3 | 16 |
| *Chortodes elymi* |  |  |  |  | 1 |  | 1 |
| *Chortodes extrema* |  |  | 6 | 1 | 1 | 36 | 44 |
| *Chortodes fluxa* |  |  | 2 |  |  |  | 2 |
| *Cidaria fulvata* | 34 | 80 | 46 |  | 39 | 13 | 212 |
| *Cilix glaucata* | 1 | 3 |  |  | 1 | 2 | 7 |
| *Cleorodes lichenaria* | 7 | 12 |  |  |  |  | 19 |
| *Clostera curtula* |  |  | 2 |  |  |  | 2 |
| *Clostera pigra* |  |  |  |  | 1 |  | 1 |
| *Colobochyla salicalis* |  |  |  | 3 |  | 2 | 5 |
| *Colocasia coryli* | 1 | 13 | 4 | 222 |  | 5 | 245 |
| *Colostygia aptata* |  | 5 |  |  |  |  | 5 |
| *Colostygia olivata* |  | 21 |  |  |  |  | 21 |
| *Colostygia pectinataria* | 31 | 673 | 108 | 862 | 3 | 4 | 1681 |
| *Comibaena bajularia* |  | 2 | 5 | 34 | 2 | 14 | 57 |
| *Coscinia cribraria* |  |  |  |  |  | 7 | 7 |
| *Cosmia affinis* |  | 1 | 2 |  |  | 1 | 4 |
| *Cosmia pyralina* |  |  | 9 | 1 | 1 |  | 11 |
| *Cosmia trapezina* | 10 | 382 | 25 | 130 |  | 91 | 638 |
| *Cosmorhoe ocellata* | 1 | 28 | 6 | 5 | 1 | 2 | 43 |
| *Cosmotriche lobulina* | 10 | 266 |  | 1 |  |  | 277 |
| *Craniophora ligustri* | 18 | 202 | 23 | 181 |  | 7 | 431 |
| *Crocallis elinguaria* | 36 | 116 | 6 | 33 |  |  | 191 |
| *Cryphia algae* | 1 | 2 |  |  | 2 | 24 | 29 |
| *Cucullia scrophulariae* |  |  |  |  |  | 1 | 1 |
| *Cucullia umbratica* | 2 |  | 1 |  |  |  | 3 |
| *Cybosia mesomella* | 7 | 5 | 17 | 6 | 5 | 73 | 113 |
| *Cyclophora albipunctata* |  |  |  |  |  | 13 | 13 |
| *Cyclophora annularia* |  | 3 |  | 33 |  |  | 36 |
| *Cyclophora linearia* | 1 | 418 | 7 | 1328 | 3 | 35 | 1792 |
| *Cyclophora punctaria* |  | 5 | 5 |  | 1 | 928 | 939 |
| *Deilephila elpenor* | 2 | 4 | 7 | 2 | 3 | 12 | 30 |
| *Deilephila porcellus* | 11 | 2 | 22 |  |  |  | 35 |
| *Deileptenia ribeata* | 1 | 552 |  | 48 |  |  | 601 |
| *Deltote bankiana* |  |  | 3 |  | 2 | 11 | 16 |
| *Deltote deceptoria* | 19 | 1 | 140 | 31 | 1 | 37 | 229 |
| *Deltote uncula* |  |  |  |  | 2 |  | 2 |
| *Dendrolimus pini* | 12 | 35 | 6 | 6 |  | 125 | 184 |
| *Diachrysia chrysitis* | 6 | 10 | 5 | 9 | 8 |  | 38 |
| *Diachrysia stenochrysis* |  |  |  |  | 2 |  | 2 |
| *Diacrisia sannio* | 11 | 2 | 14 | 5 | 3 | 9 | 44 |
| *Diaphora mendica* |  |  | 1 |  |  |  | 1 |
| *Diarsia brunnea* | 22 | 601 | 7 | 194 | 2 | 39 | 865 |
| *Diarsia mendica* | 6 | 325 | 5 | 26 |  | 4 | 366 |
| *Diarsia rubi* |  |  |  |  | 29 | 3 | 32 |
| *Drepana curvatula* |  |  |  |  | 3 | 15 | 18 |
| *Drepana falcataria* |  |  | 1 | 2 | 1 | 15 | 19 |
| *Drymonia dodonaea* |  |  |  | 7 |  |  | 7 |
| *Drymonia obliterata* | 6 | 487 | 6 | 454 |  |  | 953 |
| *Drymonia velitaris* |  |  |  |  |  | 4 | 4 |
| *Dypterygia scabriuscula* |  |  |  |  |  | 22 | 22 |
| *Dysstroma citrata* |  |  |  |  | 1 | 1 | 2 |
| *Dysstroma truncata* |  | 38 | 1 | 31 | 1 | 37 | 108 |
| *Earias clorana* |  |  | 2 |  |  |  | 2 |
| *Ecliptopera capitata* |  | 488 |  | 41 |  | 8 | 537 |
| *Ecliptopera silaceata* |  | 58 | 2 | 75 | 2 | 200 | 337 |
| *Ectropis crepuscularia* | 3 | 225 | 1 | 74 | 2 | 35 | 340 |
| *Eilema complana* | 220 | 48 | 94 | 16 | 3 | 72 | 453 |
| *Eilema depressa* | 32 | 741 |  | 43 |  | 687 | 1503 |
| *Eilema griseola* |  | 1 |  |  | 11 | 7 | 19 |
| *Eilema lurideola* | 227 | 853 | 14 | 42 | 7 | 147 | 1290 |
| *Eilema lutarella* |  |  | 1 |  | 1 | 4 | 6 |
| *Eilema sororcula* |  | 11 | 34 | 499 | 1 | 2 | 547 |
| *Elaphria venustula* | 2 |  | 1 | 2 |  | 50 | 55 |
| *Electrophaes corylata* |  | 1 |  | 4 |  |  | 5 |
| *Ematurga atomaria* |  |  | 2 |  | 6 |  | 8 |
| *Enargia paleacea* |  |  |  |  | 1 | 3 | 4 |
| *Ennomos alniaria* |  |  |  |  |  | 2 | 2 |
| *Ennomos erosaria* |  |  |  |  |  | 16 | 16 |
| *Ennomos fuscantaria* |  |  | 1 |  | 3 |  | 4 |
| *Ennomos quercinaria* | 1 | 77 |  | 163 |  | 5 | 246 |
| *Epilecta linogrisea* |  |  |  |  |  | 1 | 1 |
| *Epione repandaria* |  |  |  |  | 2 | 8 | 10 |
| *Epirrhoe alternata* | 16 | 252 | 20 | 54 | 20 | 20 | 382 |
| *Epirrhoe hastulata* |  |  | 1 |  |  |  | 1 |
| *Epirrhoe molluginata* |  | 4 |  | 12 |  |  | 16 |
| *Epirrhoe rivata* |  | 2 |  |  | 4 | 4 | 10 |
| *Epirrhoe tristata* | 19 |  | 13 |  | 13 |  | 45 |
| *Eublemma minutata* |  |  |  |  |  | 1 | 1 |
| *Eucarta virgo* |  |  |  |  | 2 | 2 | 4 |
| *Euchalcia variabilis* | 1 | 2 | 2 |  |  |  | 5 |
| *Euchoeca nebulata* |  | 1 | 4 | 1 | 3 | 1 | 10 |
| *Euclidia glyphica* |  |  | 1 |  |  | 1 | 2 |
| *Eulithis mellinata* |  |  |  |  |  | 1 | 1 |
| *Eulithis populata* | 1 | 5 | 1 |  |  | 30 | 37 |
| *Eulithis prunata* | 3 | 49 | 4 | 8 |  | 2 | 66 |
| *Euphyia biangulata* | 1 | 7 |  |  |  |  | 8 |
| *Euphyia unangulata* |  | 1 |  | 15 |  | 109 | 125 |
| *Eupithecia abbreviata* |  | 1 |  | 12 |  |  | 13 |
| *Eupithecia abietaria* |  | 3 |  | 2 |  |  | 5 |
| *Eupithecia absinthiata* |  | 2 |  |  |  |  | 2 |
| *Eupithecia centaureata* |  | 1 | 10 |  | 2 | 8 | 21 |
| *Eupithecia expallidata* |  |  |  | 1 |  |  | 1 |
| *Eupithecia haworthiata* |  |  | 1 |  |  |  | 1 |
| *Eupithecia icterata* | 2 | 1 | 1 |  |  | 4 | 8 |
| *Eupithecia innotata* |  |  | 1 |  |  |  | 1 |
| *Eupithecia inturbata* |  |  |  |  | 1 | 1 | 2 |
| *Eupithecia lariciata* |  |  |  | 3 |  |  | 3 |
| *Eupithecia plumbeolata* | 1 | 2 |  | 4 | 1 | 5 | 13 |
| *Eupithecia subfuscata* | 8 | 33 | 29 | 52 | 1 | 32 | 155 |
| *Eupithecia subumbrata* | 1 |  | 2 |  |  |  | 3 |
| *Eupithecia tantillaria* |  | 1 |  | 21 |  |  | 22 |
| *Eupithecia tenuiata* |  |  |  |  |  | 7 | 7 |
| *Eupithecia tripunctaria* |  | 2 | 1 | 3 |  | 1 | 7 |
| *Euplexia lucipara* | 5 | 56 |  | 22 |  | 14 | 97 |
| *Euproctis chrysorrhoea* |  | 1 |  |  | 1 | 16 | 18 |
| *Eurois occulta* |  | 1 |  |  |  | 2 | 3 |
| *Eustroma reticulata* |  | 67 |  | 4 |  |  | 71 |
| *Euthrix potatoria* |  |  | 7 | 36 | 2 | 5 | 50 |
| *Euxoa aquilina* |  |  |  | 2 |  |  | 2 |
| *Euxoa nigrofusca* |  |  | 1 |  |  |  | 1 |
| *Euxoa obelisca* |  |  |  |  |  | 1 | 1 |
| *Fagivorina arenaria* | 1 | 6 |  |  |  |  | 7 |
| *Falcaria lacertinaria* |  |  |  |  |  | 23 | 23 |
| *Gandaritis pyraliata* | 15 | 17 | 3 |  | 3 |  | 38 |
| *Gastropacha quercifolia* |  |  | 2 |  | 1 |  | 3 |
| *Geometra papilionaria* | 2 |  | 1 |  |  | 17 | 20 |
| *Gluphisia crenata* |  | 1 | 1 | 2 | 1 |  | 5 |
| *Gortyna flavago* |  |  |  |  | 7 |  | 7 |
| *Graphiphora augur* | 3 | 2 | 4 | 1 | 1 |  | 11 |
| *Gymnoscelis rufifasciata* |  |  |  |  |  | 3 | 3 |
| *Habrosyne pyritoides* | 26 | 73 | 13 | 9 | 10 | 63 | 194 |
| *Hada plebeja* | 3 | 23 | 38 | 1 | 4 | 1 | 70 |
| *Hadena bicruris* |  |  |  |  |  | 7 | 7 |
| *Hadena capsincola* |  |  |  |  | 1 | 1 | 2 |
| *Hadena perplexa* |  |  |  |  |  | 2 | 2 |
| *Hecatera bicolorata* |  |  |  | 1 |  |  | 1 |
| *Heliothis viriplaca* |  |  | 3 |  | 6 | 1 | 10 |
| *Hemistola chrysoprasaria* |  | 1 | 2 | 3 |  |  | 6 |
| *Hemithea aestivaria* |  | 27 | 9 | 14 |  | 19 | 69 |
| *Hepialus humuli* | 4 |  | 1 | 10 |  | 1 | 16 |
| *Herminia grisealis* | 6 | 21 | 1 | 111 |  | 8 | 147 |
| *Herminia tarsicrinalis* | 5 | 87 | 4 | 6 | 1 | 16 | 119 |
| *Heterogenea asella* |  | 2 |  |  |  |  | 2 |
| *Hoplodrina ambigua* | 2 | 3 | 2 |  | 17 | 204 | 228 |
| *Hoplodrina blanda* | 459 | 874 | 267 | 53 | 6 | 9 | 1668 |
| *Hoplodrina octogenaria* | 96 | 129 | 76 | 27 | 18 | 20 | 366 |
| *Hoplodrina respersa* | 7 | 17 |  |  | 1 |  | 25 |
| *Horisme radicaria* |  |  |  | 6 |  | 1 | 7 |
| *Horisme tersata* |  |  |  | 2 |  |  | 2 |
| *Horisme vitalbata* |  |  | 1 |  |  |  | 1 |
| *Hydraecia micacea* | 3 |  | 2 |  | 40 | 1 | 46 |
| *Hydrelia flammeolaria* |  | 64 |  | 68 | 3 | 17 | 152 |
| *Hydrelia sylvata* |  | 9 |  | 13 |  |  | 22 |
| *Hydriomena furcata* | 90 | 745 | 13 | 91 | 3 | 4 | 946 |
| *Hydriomena impluviata* |  | 1 |  | 12 |  |  | 13 |
| *Hylaea fasciaria* |  | 102 | 2 | 67 |  | 74 | 245 |
| *Hyles euphorbiae* |  |  | 9 |  | 1 |  | 10 |
| *Hyles galii* |  |  |  | 1 |  |  | 1 |
| *Hyloicus pinastri* |  | 10 | 7 | 5 |  | 24 | 46 |
| *Hypena crassalis* |  | 2 |  |  |  | 14 | 16 |
| *Hypena proboscidalis* | 21 | 1051 | 8 | 208 | 6 | 104 | 1398 |
| *Hypomecis punctinalis* | 3 | 167 | 13 | 868 | 4 | 248 | 1303 |
| *Hypomecis roboraria* | 3 | 442 | 6 | 1092 |  | 394 | 1937 |
| *Idaea aversata* | 5 | 648 | 26 | 360 | 6 | 93 | 1138 |
| *Idaea biselata* | 3 | 233 | 1 | 65 |  | 12 | 314 |
| *Idaea dimidiata* |  |  |  | 1 |  | 1 | 2 |
| *Idaea emarginata* |  |  | 1 |  |  |  | 1 |
| *Idaea humiliata* | 35 |  | 45 |  |  | 1 | 81 |
| *Idaea muricata* |  |  | 5 |  |  |  | 5 |
| *Idaea ochrata* |  |  | 7 |  |  | 2 | 9 |
| *Idaea rufaria* |  |  | 1 |  |  |  | 1 |
| *Idaea straminata* |  |  |  |  |  | 2 | 2 |
| *Idia calvaria* |  | 7 |  |  |  |  | 7 |
| *Ipimorpha subtusa* | 1 | 1 |  | 1 |  |  | 3 |
| *Jodis lactearia* |  | 2 |  | 24 |  |  | 26 |
| *Korscheltellus lupulina* |  | 12 | 3 | 73 |  |  | 88 |
| *Lacanobia oleracea* | 1 |  | 2 | 2 | 9 | 19 | 33 |
| *Lacanobia splendens* |  |  |  |  | 7 | 5 | 12 |
| *Lacanobia suasa* |  |  |  | 1 | 1 |  | 2 |
| *Lacanobia thalassina* | 5 | 85 | 16 | 34 | 1 | 25 | 166 |
| *Lacanobia w-latinum* |  |  | 7 |  |  | 3 | 10 |
| *Lampropteryx suffumata* |  |  |  |  |  | 1 | 1 |
| *Laothoe populi* | 1 | 2 | 1 | 3 | 5 | 2 | 14 |
| *Lasiocampa quercus* |  |  |  | 2 |  |  | 2 |
| *Lasiocampa trifolii* |  |  |  |  | 1 |  | 1 |
| *Laspeyria flexula* | 11 | 1802 | 45 | 556 | 1 | 55 | 2470 |
| *Leucania comma* |  |  | 2 |  |  |  | 2 |
| *Leucania obsoleta* |  |  |  |  | 4 | 3 | 7 |
| *Leucodonta bicoloria* |  |  | 1 | 24 |  | 10 | 35 |
| *Ligdia adustata* |  | 7 |  | 1 | 3 | 6 | 17 |
| *Lithomoia solidaginis* |  |  |  |  |  | 1 | 1 |
| *Lithosia quadra* | 32 | 1216 |  | 31 |  | 48 | 1327 |
| *Lomaspilis marginata* | 13 | 105 | 26 | 35 | 33 | 77 | 289 |
| *Lomographa bimaculata* |  |  | 3 | 15 |  | 1 | 19 |
| *Lomographa temerata* | 3 | 47 | 26 | 69 |  | 7 | 152 |
| *Luperina testacea* |  |  |  |  | 8 | 1 | 9 |
| *Lycophotia porphyrea* |  |  |  |  |  | 3 | 3 |
| *Lygephila pastinum* | 8 | 3 | 5 | 5 |  | 1 | 22 |
| *Lygephila viciae* |  | 1 |  |  |  | 1 | 2 |
| *Lymantria dispar* |  | 1 |  |  |  |  | 1 |
| *Lymantria monacha* | 3 | 2036 |  | 289 |  | 17 | 2345 |
| *Lythria purpuraria* |  |  |  |  | 7 |  | 7 |
| *Macaria alternata* | 4 | 35 | 19 | 13 | 8 | 77 | 156 |
| *Macaria brunneata* |  |  | 5 | 1 | 1 | 21 | 28 |
| *Macaria liturata* | 1 | 524 | 11 | 116 | 5 | 854 | 1511 |
| *Macaria notata* |  |  |  |  |  | 9 | 9 |
| *Macaria signaria* |  | 75 | 5 | 97 |  | 5 | 182 |
| *Macaria wauaria* | 11 | 69 | 18 | 7 |  | 45 | 150 |
| *Macdunnoughia confusa* |  |  |  |  |  | 3 | 3 |
| *Macrochilo cribrumalis* |  |  |  |  | 1 |  | 1 |
| *Macrothylacia rubi* | 2 |  | 8 | 1 |  |  | 11 |
| *Malacosoma castrensis* | 36 | 1 | 8 |  |  |  | 45 |
| *Malacosoma neustria* | 176 | 199 | 296 | 6 | 71 | 36 | 784 |
| *Mamestra brassicae* | 1 |  | 3 | 1 | 3 | 4 | 12 |
| *Martania taeniata* |  |  |  |  |  | 3 | 3 |
| *Meganola albula* |  |  | 1 | 1 |  |  | 2 |
| *Meganola strigula* |  |  |  |  |  | 7 | 7 |
| *Meganola togatulalis* |  |  |  |  |  | 1 | 1 |
| *Melanchra persicariae* | 6 | 42 |  | 5 |  | 2 | 55 |
| *Melanthia procellata* |  |  |  | 5 |  | 1 | 6 |
| *Mesapamea didyma* | 2 | 65 | 4 | 17 | 2 | 10 | 100 |
| *Mesapamea secalis* | 2 | 18 |  |  |  | 13 | 33 |
| *Mesoleuca albicillata* |  | 26 |  | 8 |  | 68 | 102 |
| *Mesoligia furuncula* | 18 | 6 | 59 |  | 14 | 45 | 142 |
| *Mesotype didymata* |  | 1 |  |  |  | 1 | 2 |
| *Miltochrista miniata* |  | 1 |  |  | 7 | 57 | 65 |
| *Mimas tiliae* |  |  | 2 |  | 1 |  | 3 |
| *Minoa murinata* |  |  |  |  |  | 1 | 1 |
| *Moma alpium* |  | 9 |  | 44 |  | 6 | 59 |
| *Mythimna albipuncta* | 5 | 4 | 4 |  | 8 | 27 | 48 |
| *Mythimna conigera* | 81 | 4 | 35 |  | 7 | 1 | 128 |
| *Mythimna ferrago* | 30 | 23 | 23 | 3 |  | 1 | 80 |
| *Mythimna impura* | 56 | 28 | 34 | 3 | 65 | 14 | 200 |
| *Mythimna pallens* | 7 |  | 104 |  | 200 | 66 | 377 |
| *Mythimna pudorina* | 4 |  | 2 |  | 39 | 5 | 50 |
| *Mythimna scirpi* |  |  | 2 | 1 |  |  | 3 |
| *Mythimna sicula* |  |  |  |  |  | 1 | 1 |
| *Mythimna turca* |  |  |  |  | 1 | 2 | 3 |
| *Mythimna vitellina* | 2 | 1 |  |  |  |  | 3 |
| *Noctua comes* | 1 |  | 2 | 1 | 1 |  | 5 |
| *Noctua fimbriata* | 12 | 1 | 7 |  | 5 | 38 | 63 |
| *Noctua interposita* |  |  | 5 |  | 2 | 2 | 9 |
| *Noctua janthe* | 1 | 4 |  | 3 | 1 | 8 | 17 |
| *Noctua janthina* | 1 |  | 1 |  |  |  | 2 |
| *Noctua orbona* |  |  | 3 |  |  | 67 | 70 |
| *Noctua pronuba* | 14 | 26 | 6 | 4 | 14 | 71 | 135 |
| *Nola cristatula* |  |  | 2 |  | 1 | 3 | 6 |
| *Nola cucullatella* |  |  | 1 |  |  |  | 1 |
| *Notodonta dromedarius* | 2 |  |  |  |  | 11 | 13 |
| *Notodonta ziczac* | 1 | 2 |  |  |  |  | 3 |
| *Nycteola asiatica* |  |  |  |  |  | 3 | 3 |
| *Nycteola revayana* |  | 2 | 1 | 2 |  | 1 | 6 |
| *Ochropacha duplaris* | 6 | 42 |  | 1 |  | 10 | 59 |
| *Ochropleura plecta* | 44 | 52 | 62 | 49 | 77 | 84 | 368 |
| *Odezia atrata* |  |  | 8 |  |  |  | 8 |
| *Odontopera bidentata* |  |  |  | 2 |  |  | 2 |
| *Oligia fasciuncula* |  |  | 71 | 2 | 3 | 1 | 77 |
| *Oligia latruncula* | 101 | 169 | 246 | 57 | 24 | 15 | 612 |
| *Oligia strigilis* | 132 | 61 | 86 | 29 | 18 | 8 | 334 |
| *Oligia versicolor* | 20 | 122 | 7 | 15 | 3 | 5 | 172 |
| *Opisthograptis luteolata* |  | 1 | 3 | 4 |  |  | 8 |
| *Orgyia antiqua* |  | 1 | 1 | 4 | 1 | 5 | 12 |
| *Orgyia antiquiodes* |  |  |  |  | 1 |  | 1 |
| *Orthonama vittata* |  |  |  |  | 14 |  | 14 |
| *Ourapteryx sambucaria* |  | 4 |  |  |  |  | 4 |
| *Pachetra sagittigera* |  |  | 1 |  |  |  | 1 |
| *Panthea coenobita* | 6 | 183 | 3 | 7 |  | 2 | 201 |
| *Paracolax tristalis* |  |  |  |  |  | 9 | 9 |
| *Parascotia fuliginaria* |  | 2 |  | 2 |  | 4 | 8 |
| *Parectropis similaria* |  | 27 | 4 | 303 |  | 39 | 373 |
| *Pasiphila debiliata* |  | 10 |  | 5 |  | 18 | 33 |
| *Pasiphila rectangulata* | 5 | 2 | 5 | 1 | 3 |  | 16 |
| *Pechipogo strigilata* | 3 | 1 |  |  |  | 2 | 6 |
| *Pelosia muscerda* |  |  |  |  |  | 17 | 17 |
| *Pelurga comitata* |  |  |  |  |  | 4 | 4 |
| *Pennithera firmata* |  |  |  |  |  | 1 | 1 |
| *Perconia strigillaria* |  |  | 1 |  |  |  | 1 |
| *Peribatodes rhomboidaria* | 4 | 46 | 3 |  | 2 | 42 | 97 |
| *Peribatodes secundaria* |  | 97 |  | 14 |  | 9 | 120 |
| *Perizoma affinitata* |  | 3 |  |  |  | 1 | 4 |
| *Perizoma albulata* | 1 | 3 | 3 | 1 |  |  | 8 |
| *Perizoma alchemillata* | 54 | 381 | 10 | 7 | 21 | 716 | 1189 |
| *Perizoma blandiata* | 2 |  |  |  |  |  | 2 |
| *Perizoma hydrata* |  | 1 |  |  |  |  | 1 |
| *Petrophora chlorosata* |  |  |  |  |  | 10 | 10 |
| *Phalera bucephala* | 4 | 179 | 8 | 247 | 1 | 25 | 464 |
| *Pheosia gnoma* |  |  |  |  | 3 | 47 | 50 |
| *Pheosia tremula* |  |  | 5 | 1 | 2 | 6 | 14 |
| *Phibalapteryx virgata* | 29 |  |  |  |  |  | 29 |
| *Philereme transversata* |  | 19 |  | 1 |  |  | 20 |
| *Philereme vetulata* | 5 | 17 | 4 | 1 | 5 | 12 | 44 |
| *Phlogophora meticulosa* | 1 | 1 |  | 2 |  |  | 4 |
| *Phlogophora scita* |  | 20 |  |  |  |  | 20 |
| *Photedes minima* | 3 |  | 1 | 9 |  |  | 13 |
| *Phragmataecia castaneae* |  |  |  |  | 15 | 6 | 21 |
| *Phragmatobia fuliginosa* | 86 | 5 | 275 | 10 | 27 | 6 | 409 |
| *Phymatopus hecta* |  | 18 |  | 4 |  | 14 | 36 |
| *Phytometra viridaria* | 4 |  |  |  |  |  | 4 |
| *Plagodis dolabraria* |  | 4 | 1 | 13 |  | 53 | 71 |
| *Plemyria rubiginata* | 9 | 17 | 8 | 2 |  |  | 36 |
| *Plusia festucae* |  |  | 1 |  | 7 | 7 | 15 |
| *Poecilocampa populi* | 1 |  |  |  |  |  | 1 |
| *Polia bombycina* |  |  | 3 |  |  |  | 3 |
| *Polia nebulosa* | 10 | 64 | 2 | 22 |  | 12 | 110 |
| *Polymixis gemmea* |  |  |  |  |  | 7 | 7 |
| *Protodeltote pygarga* | 9 | 439 | 12 | 150 | 2 | 386 | 998 |
| *Pseudeustrotia candidula* |  |  |  |  | 73 | 15 | 88 |
| *Pseudoips prasinana* |  | 9 | 4 | 56 | 1 | 21 | 91 |
| *Pseudopanthera macularia* |  |  |  | 1 |  |  | 1 |
| *Pterostoma palpina* |  |  | 1 |  |  |  | 1 |
| *Ptilodon capucina* | 2 | 83 | 3 | 64 | 1 | 18 | 171 |
| *Ptilodon cucullina* | 5 | 111 | 1 | 14 | 1 |  | 132 |
| *Pungeleria capreolaria* |  | 1 |  | 6 |  | 1 | 8 |
| *Pyrrhia umbra* | 7 |  | 5 |  |  |  | 12 |
| *Rheumaptera undulata* |  | 3 |  | 1 |  | 4 | 8 |
| *Rhodostrophia vibicaria* |  | 1 |  | 1 |  | 1 | 3 |
| *Rhyparia purpurata* |  |  | 3 |  | 2 | 1 | 6 |
| *Rivula sericealis* | 19 | 50 | 43 | 40 | 45 | 144 | 341 |
| *Rusina ferruginea* | 46 | 61 | 13 | 6 | 3 | 54 | 183 |
| *Sabra harpagula* |  |  |  |  | 1 | 4 | 5 |
| *Schrankia taenialis* |  |  |  |  |  | 1 | 1 |
| *Scopula floslactata* |  | 1 | 2 | 2 |  |  | 5 |
| *Scopula immorata* | 18 |  | 44 |  | 3 | 4 | 69 |
| *Scopula immutata* |  |  | 2 | 1 | 28 | 12 | 43 |
| *Scopula nigropunctata* | 1 | 18 | 2 | 3 | 5 | 502 | 531 |
| *Scopula ornata* | 5 |  |  |  |  |  | 5 |
| *Scopula rubiginata* |  |  | 9 |  | 35 | 19 | 63 |
| *Scotopteryx bipunctaria* | 3 |  |  |  |  |  | 3 |
| *Scotopteryx chenopodiata* | 35 | 15 | 41 | 20 | 8 |  | 119 |
| *Scotopteryx mucronata* | 1 |  | 1 |  |  |  | 2 |
| *Selenia dentaria* | 4 | 84 | 1 | 70 |  |  | 159 |
| *Selenia lunularia* |  |  |  | 29 |  |  | 29 |
| *Selenia tetralunaria* | 4 | 220 | 2 | 72 |  |  | 298 |
| *Setina irrorella* | 4 |  | 8 |  |  |  | 12 |
| *Sideridis reticulata* | 5 |  | 1 |  | 5 | 2 | 13 |
| *Sideridis rivularis* | 1 |  |  |  |  | 1 | 2 |
| *Siona lineata* |  |  | 12 | 2 |  |  | 14 |
| *Smerinthus ocellata* |  |  | 3 |  |  | 2 | 5 |
| *Spargania luctuata* |  |  |  | 1 |  |  | 1 |
| *Sphinx ligustri* | 3 | 2 | 5 | 3 |  |  | 13 |
| *Spilosoma lubricipeda* | 6 | 10 | 22 | 30 | 19 | 29 | 116 |
| *Spilosoma lutea* | 3 | 22 | 3 | 65 | 17 | 114 | 224 |
| *Stauropus fagi* | 2 | 53 | 7 | 170 |  | 11 | 243 |
| *Stegania cararia* |  |  |  | 1 |  |  | 1 |
| *Tethea ocularis* |  |  | 1 |  |  | 1 | 2 |
| *Tethea or* |  | 9 | 1 | 2 | 1 | 8 | 21 |
| *Tetheella fluctuosa* | 1 | 4 |  |  |  | 3 | 8 |
| *Thalera fimbrialis* |  |  | 7 |  |  |  | 7 |
| *Thalpophila matura* |  |  |  |  | 1 | 11 | 12 |
| *Thaumetopoea processionea* | 22 | 2 |  |  | 1 | 5 | 30 |
| *Thera britannica* |  | 3 |  |  |  |  | 3 |
| *Thera obeliscata* | 3 | 6 | 6 | 13 | 1 | 5 | 34 |
| *Thera variata* | 1 | 8 |  | 4 |  |  | 13 |
| *Thera vetustata* |  | 1 |  |  |  |  | 1 |
| *Thumatha senex* |  |  |  | 2 | 10 | 1 | 13 |
| *Thyatira batis* | 1 | 60 |  | 9 | 1 | 43 | 114 |
| *Timandra comae* | 1 | 12 | 2 | 15 | 13 | 519 | 562 |
| *Trachea atriplicis* |  | 4 | 1 | 7 | 7 | 14 | 33 |
| *Triodia sylvina* |  |  |  |  | 7 |  | 7 |
| *Triphosa dubitata* | 1 | 1 |  |  |  |  | 2 |
| *Trisateles emortualis* | 4 | 48 |  | 51 | 2 | 2 | 107 |
| *Tyta luctuosa* |  |  | 77 | 1 |  |  | 78 |
| *Venusia blomeri* | 1 | 97 |  | 17 |  |  | 115 |
| *Watsonalla binaria* |  |  | 6 |  | 5 | 28 | 39 |
| *Watsonalla cultraria* | 12 | 133 | 5 | 212 | 5 | 328 | 695 |
| *Xanthorhoe biriviata* |  | 13 |  | 2 | 1 | 2 | 18 |
| *Xanthorhoe designata* |  | 5 |  | 4 |  | 2 | 11 |
| *Xanthorhoe ferrugata* |  | 32 | 1 | 4 |  | 11 | 48 |
| *Xanthorhoe fluctuata* | 2 | 2 | 1 | 7 |  |  | 12 |
| *Xanthorhoe montanata* | 4 | 87 | 12 | 831 | 1 | 1 | 936 |
| *Xanthorhoe quadrifasiata* | 2 | 61 |  | 41 |  | 4 | 108 |
| *Xanthorhoe spadicearia* | 25 | 86 | 47 | 28 | 10 | 54 | 250 |
| *Xestia ashworthii* |  | 1 |  |  |  |  | 1 |
| *Xestia baja* | 2 | 16 | 1 |  |  | 48 | 67 |
| *Xestia c-nigrum* | 75 | 23 | 85 | 49 | 280 | 946 | 1458 |
| *Xestia ditrapezium* | 26 | 83 | 2 | 3 |  |  | 114 |
| *Xestia sexstrigata* |  |  | 1 |  | 226 | 6 | 233 |
| *Xestia stigmatica* |  | 4 |  |  |  | 5 | 9 |
| *Xestia triangulum* | 43 | 522 | 36 | 74 | 14 | 63 | 752 |
| *Zanclognatha tarsipennalis* | 1 | 4 |  | 2 |  |  | 7 |
| *Zeuzera pyrina* |  | 1 | 4 |  |  |  | 5 |
| *Total* | 4344 | 30406 | 5160 | 15879 | 2913 | 12686 | 71388 |

**Table S5.** Results of the alpha diversity analysis. Diversity of moths was calculated for Hill numbers 0, 1, 2, and for a coverage of 70% and 90%. Significance level (**p* < 0.05, ***p* < 0.01, ****p* < 0.001) for a given predictor variable. Bold values (*p* < 0.05) indicate statistically significant results. Predictors include habitat type (grassland vs. forest), plot-scale land-use intensity, plant diversity, temperature and rainfall as measurement of seasonal climatic conditions, artificial light at night (ALAN), and landscape-scale land-use intensity.

| **Variable**  **Coverage** | **Species richness (q=0)** | | **Shannon diversity (q=1)** | | **Simpson diversity (q=2)** | |
| --- | --- | --- | --- | --- | --- | --- |
|  | **70%** | **90%** | **70%** | **90%** | **70%** | **90%** |
| **Habitat: Grassland** | -0.051 NS | **-0.148 *** | -0.087 NS | -0.121 NS | **-0.149 *** | **-0.196 **** |
| **Land use intensity** | 0.039 NS | 0.045 NS | 0.033 NS | 0.035 NS | 0.018 NS | 0.012 NS |
| **Plant diversity** | 0.050 NS | **0.068 **** | **0.054 *** | **0.064 **** | **0.058 *** | **0.064 **** |
| **Temperature** | **0.312 ***** | **0.301 ***** | **0.299 ***** | **0.311 ***** | **0.277 ***** | **0.291 ***** |
| **Rain** | 0.095 NS | 0.124 NS | 0.096 NS | 0.104 NS | 0.095 NS | 0.105 NS |
| **ALAN** | -0.042 NS | -0.024 NS | -0.050 NS | -0.053 NS | -0.072 NS | **-0.082 *** |
| **Amount of grassland** | **-0.128 ***** | **-0.111 **** | **-0.104 **** | **-0.097 **** | -0.061 NS | -0.045 NS |
| **Amount of arable field** | -0.003 NS | -0.042 NS | -0.002 NS | -0.014 NS | -0.004 NS | -0.004 NS |

**Table S6.** Results of the sensitivity analysis including all plots (without excluding those with low moth abundance). Diversity of moths was calculated for Hill numbers 0 (species richness), 1 (Shannon diversity), and 2 (Simpson diversity) at coverage levels of 70% and 90%. Significance levels are indicated as follows: **p* < 0.05, ***p* < 0.01, ****p* < 0.001. Bold values (*p* < 0.05) indicate statistically significant results. Predictors include habitat type (grassland vs. forest), plot-scale land-use intensity, plant diversity, temperature and rainfall as measurement of seasonal climatic conditions, artificial light at night (ALAN), and landscape-scale land-use intensity. This analysis complements the main results (Table S5) and assesses the robustness of findings when including all available data. Variables that lost significance in this analysis are highlighted in red, while those that became significant are shown in green. Variables that remained unchanged are in yellow.

| **Variable**  **Coverage** | **Species richness (q=0)** | | **Shannon diversity (q=1)** | | **Simpson diversity (q=2)** | |
| --- | --- | --- | --- | --- | --- | --- |
|  | **70%** | **90%** | **70%** | **90%** | **70%** | **90%** |
| **Habitat: Grassland** | -0.033 NS | -0.123 NS | -0.072 NS | -0.101 NS | -0.136 NS | **-0.178 *** |
| **Land use intensity** | 0.045 NS | **0.053 *** | 0.039 NS | 0.042 NS | 0.025 NS | 0.020 NS |
| **Plant diversity** | 0.049 NS | **0.067 *** | **0.052 *** | **0.062 *** | **0.056 *** | **0.063 *** |
| **Temperature** | **0.297 ***** | **0.287 ***** | **0.287 ***** | **0.294 ***** | **0.266 ***** | **0.278 ***** |
| **Rain** | 0.124 NS | **0.157 *** | 0.123 NS | 0.139 NS | 0.121 NS | 0.140 NS |
| **ALAN** | -0.034 NS | -0.011 NS | -0.043 NS | -0.044 NS | -0.065 NS | -0.074 NS |
| **Amount of grassland** | **-0.150 ***** | **-0.137 ***** | **-0.123 ***** | **-0.120 ***** | **-0.078 *** | -0.065 NS |
| **Amount of arable field** | -0.006 NS | -0.044 NS | -0.004 NS | -0.016 NS | -0.006 NS | -0.006 NS |


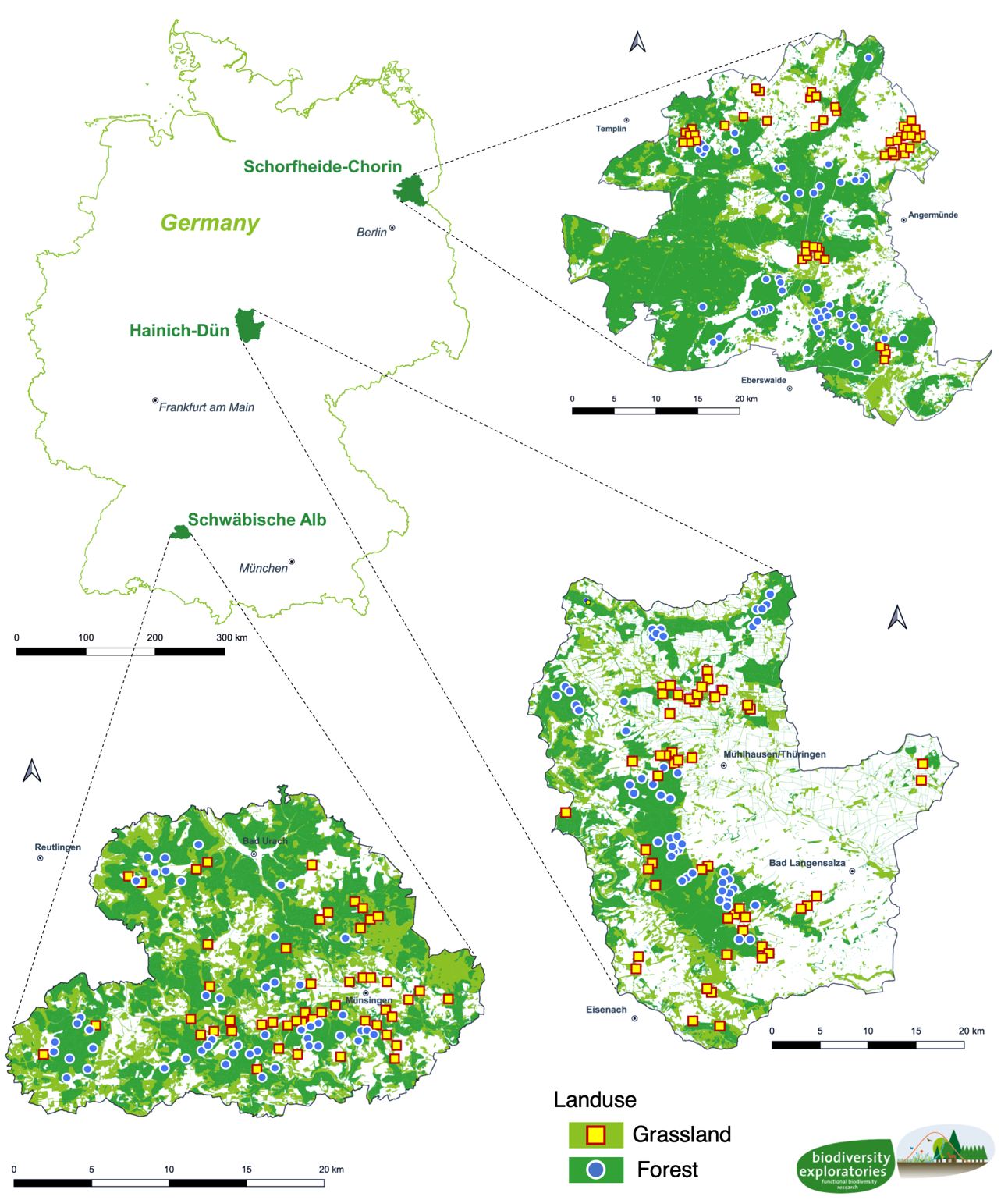


**Figure S1.** The study was conducted in three regions of Germany: Schorfheide-Chorin, Hainich-Dün, and Schwäbische Alb. In each region, 50 grassland plots (yellow squares) and 50 forest plots (blue circles) were established. The maps show the boundaries of each exploratory, with grassland areas depicted in light green and forest areas in dark green. Map created from <https://www.bexis.uni-jena.de> (Dataset ID= 31151; Memmert 2023)


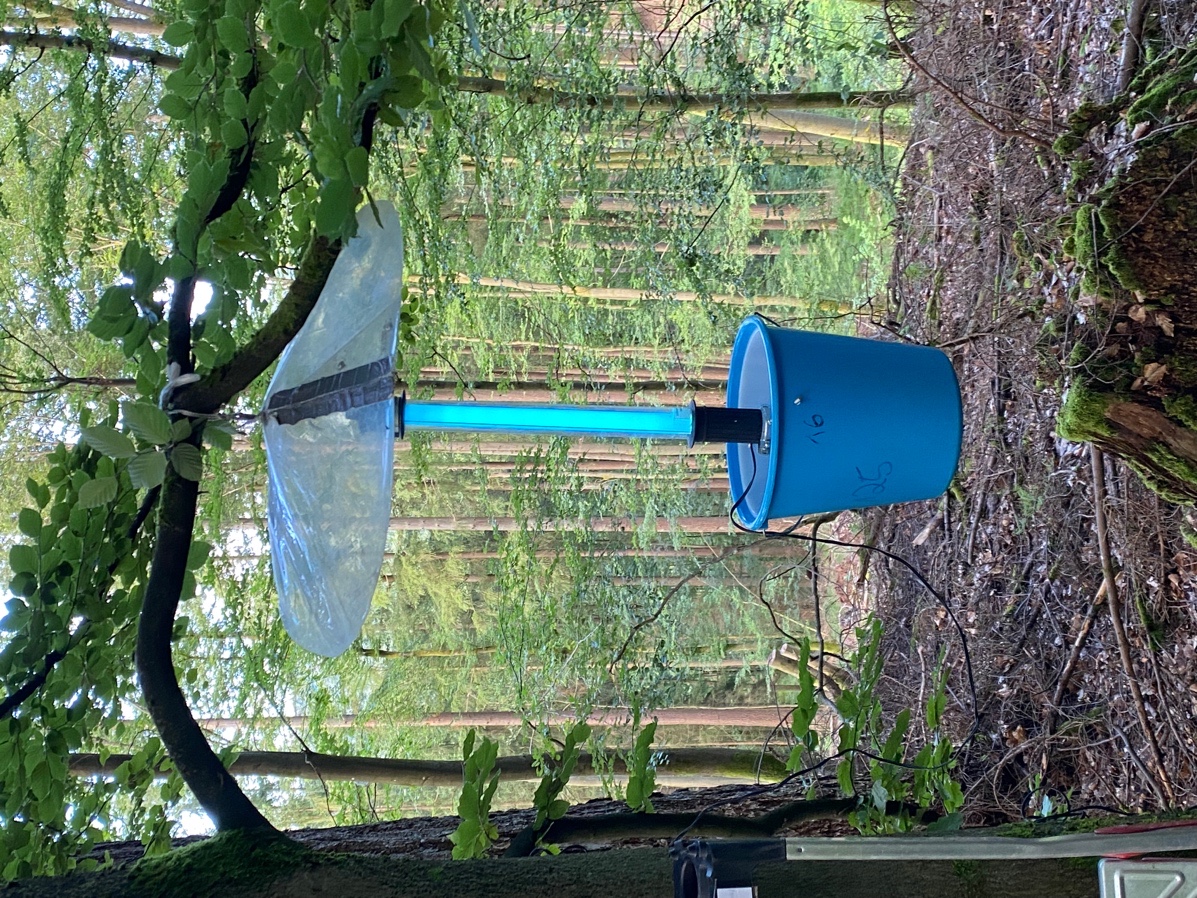


**Figure S2.** Portable automated light traps located in a forest plot. Traps run with a 15 Watt UV-Light powered by a 12 Volt battery. The traps were hung in a standardized height at around 1.5-2 m, without vegetation close by to ensure a free approach. Moths were collected in a bucket with funnel opening 6 cm diameter and chloroform as killing agent.


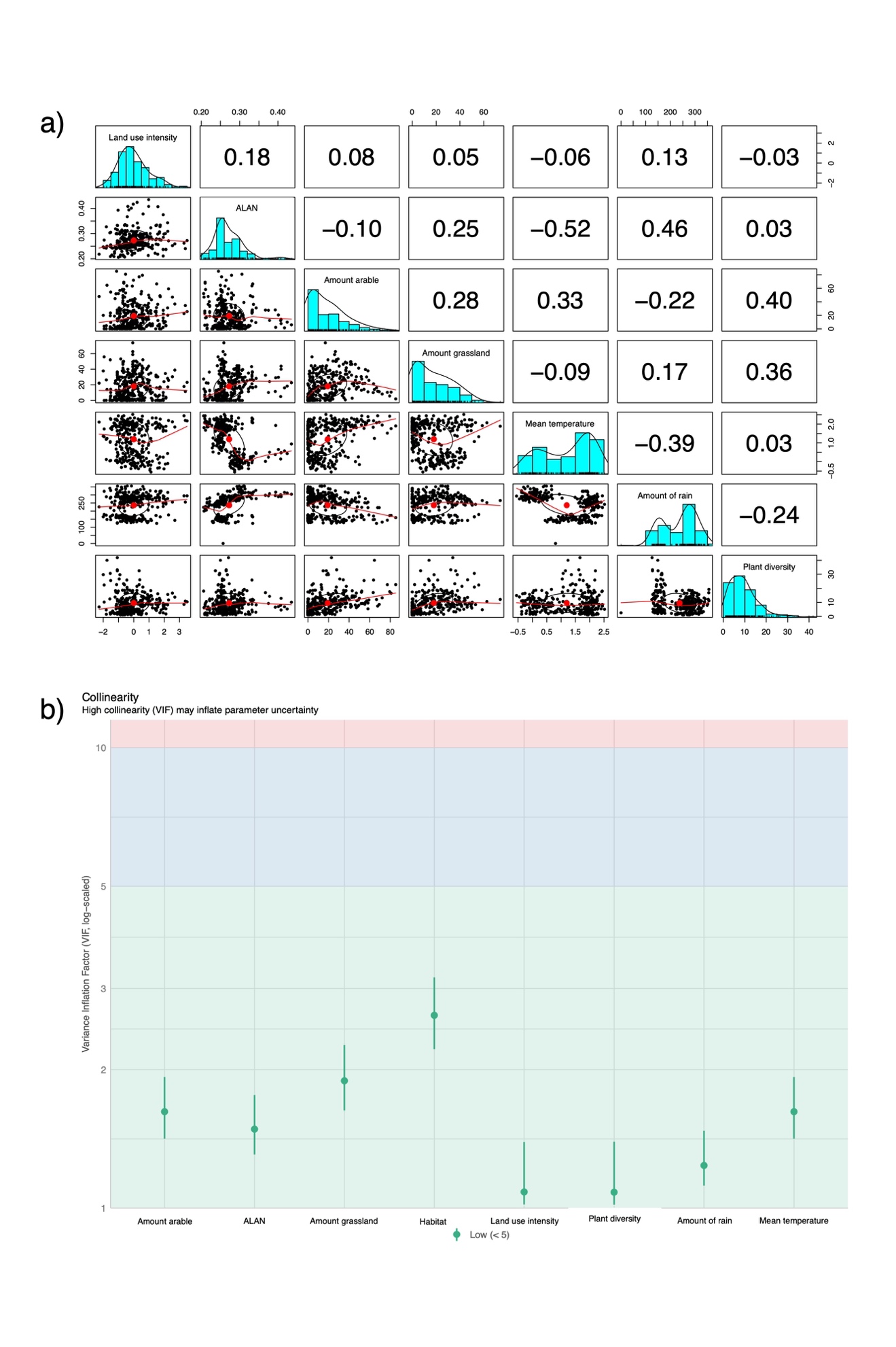


**Figure S3.** Collinearity based on correlation matrix (a) and values of the variance inflation factor (VIF) (b) used to evaluate how moth diversity was affected by the predictor variables associated with habitat, plot- and landscape-scale land-use intensity and seasonal weather conditions.


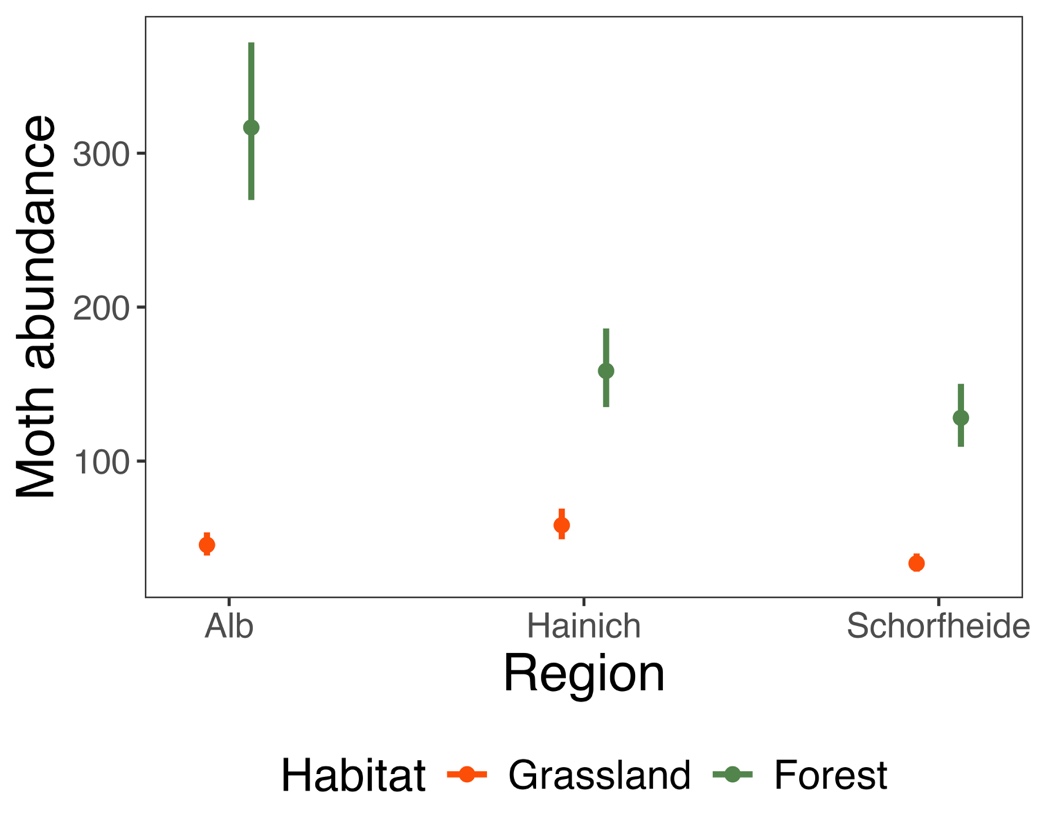


**Figure S4.** Number of moth individuals across three regions in Germany (Alb, Hainich, and Schorfheide) for two distinct habitats: grassland and forest. The data points represent mean values, while the error bars indicate variability based on confidence intervals.


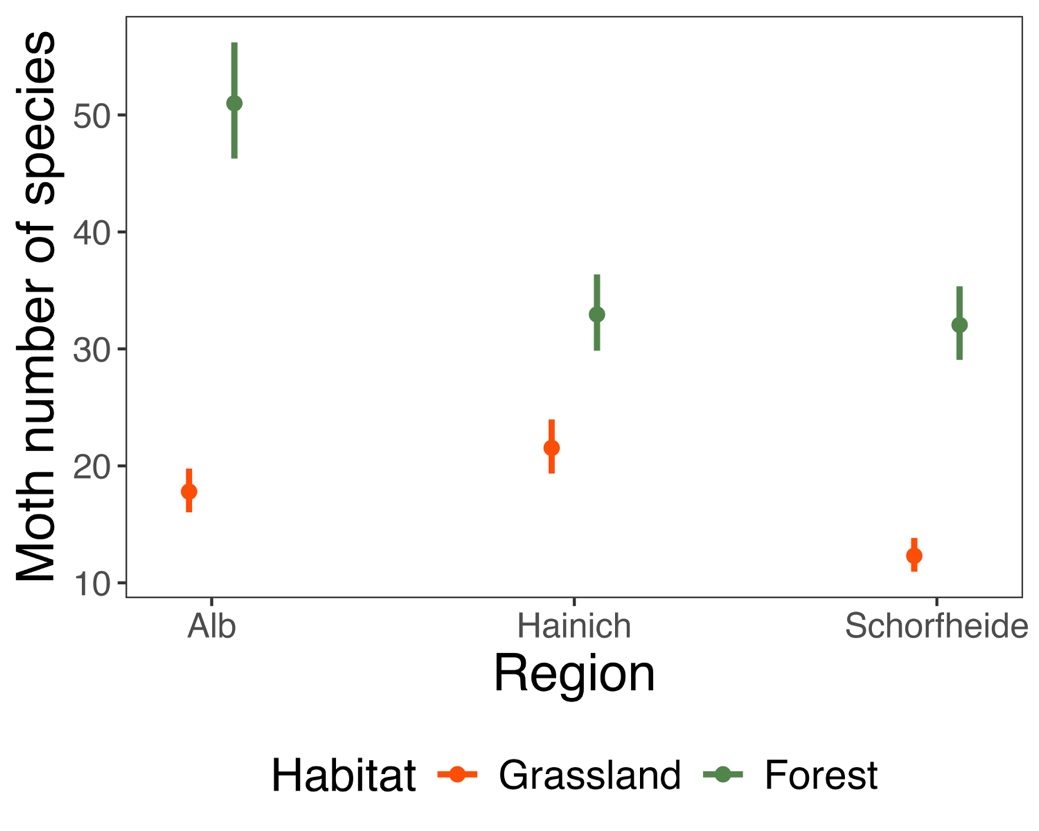


**Figure S5.** Number of moth species across three regions in Germany (Alb, Hainich, and Schorfheide) for two distinct habitats: grassland and forest. The data points represent mean values, while the error bars indicate variability based on confidence intervals.


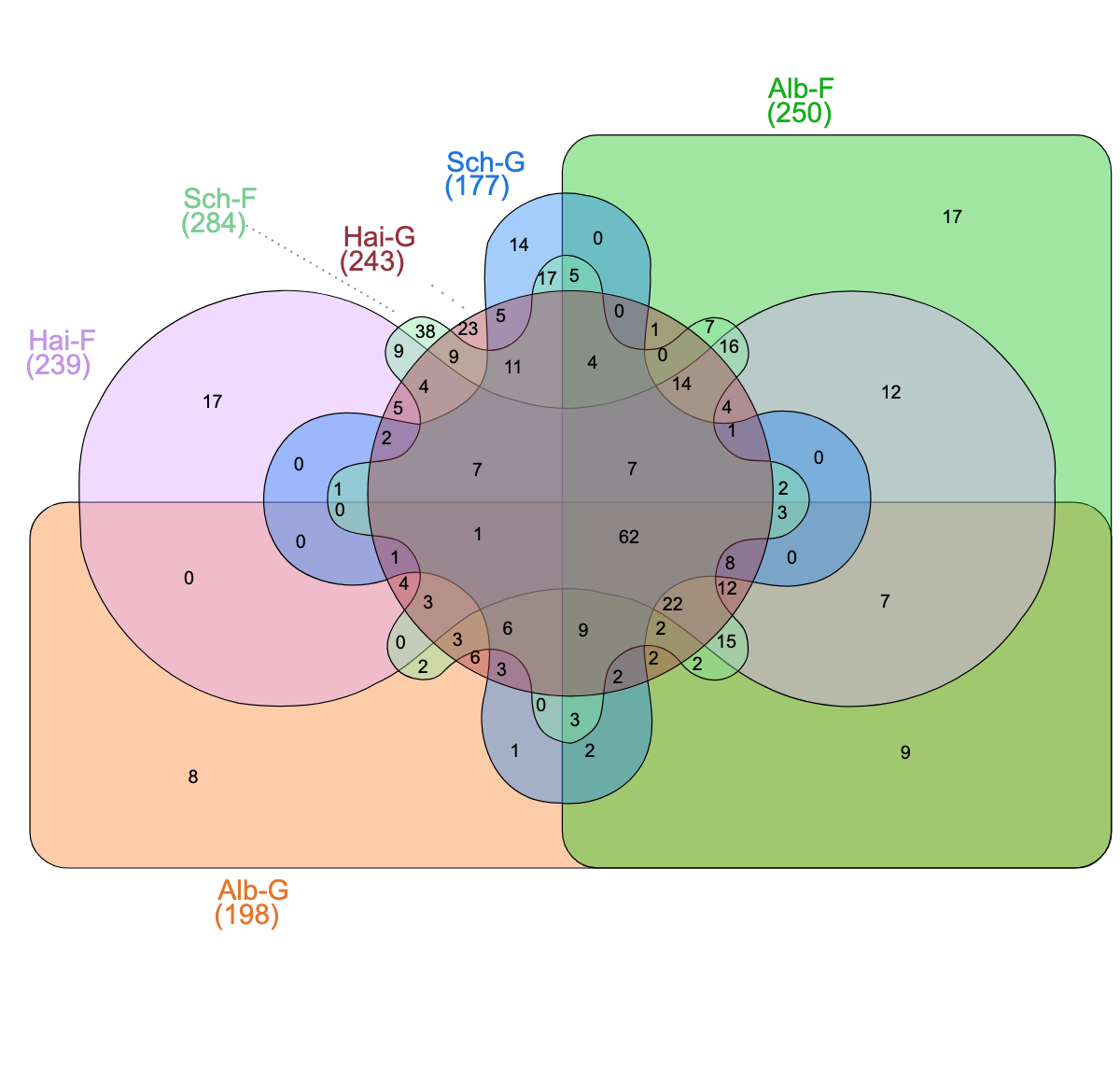
**Figure S6.** Number of species detected in each of the habitats per region (number in parentheses). The Venn diagram shows the species shared among habitats (intersection of circles). Heberle, H.; Meirelles, G. V.; da Silva, F. R.; Telles, G. P.; Minghim, R. InteractiVenn: a web-based tool for the analysis of sets through Venn diagrams. BMC Bioinformatics 16:169 (2015). https://www.interactivenn.net/index2.html


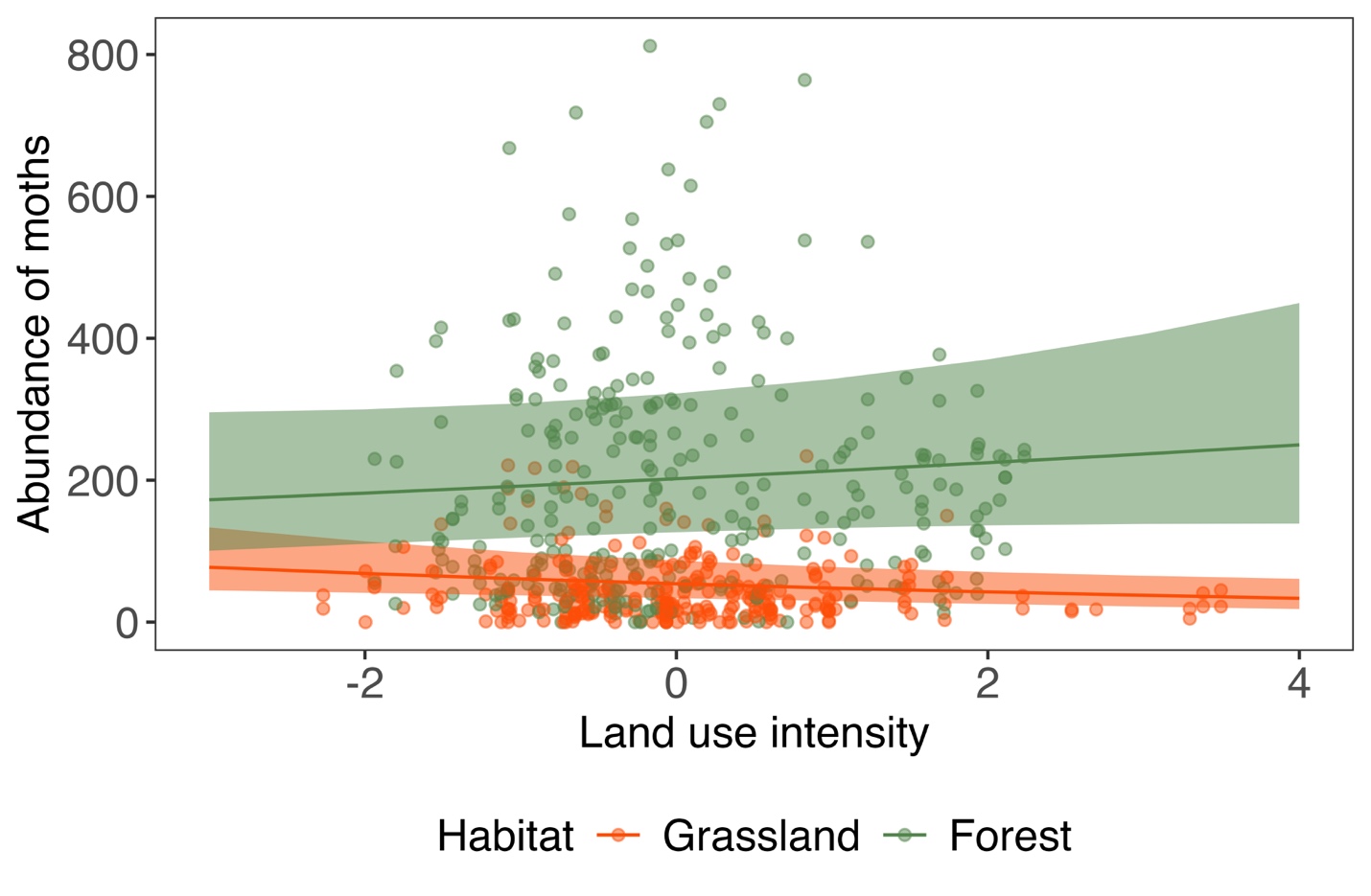
**Figure S7.** Effect of the land use intensity and its interaction with habitat on moth abundance captured in 584 traps-nights. Dots are raw data while significant (*p* < 0.05) relationships predicted by glmm with negative binomial family are indicated with a solid line (± 95% CI).


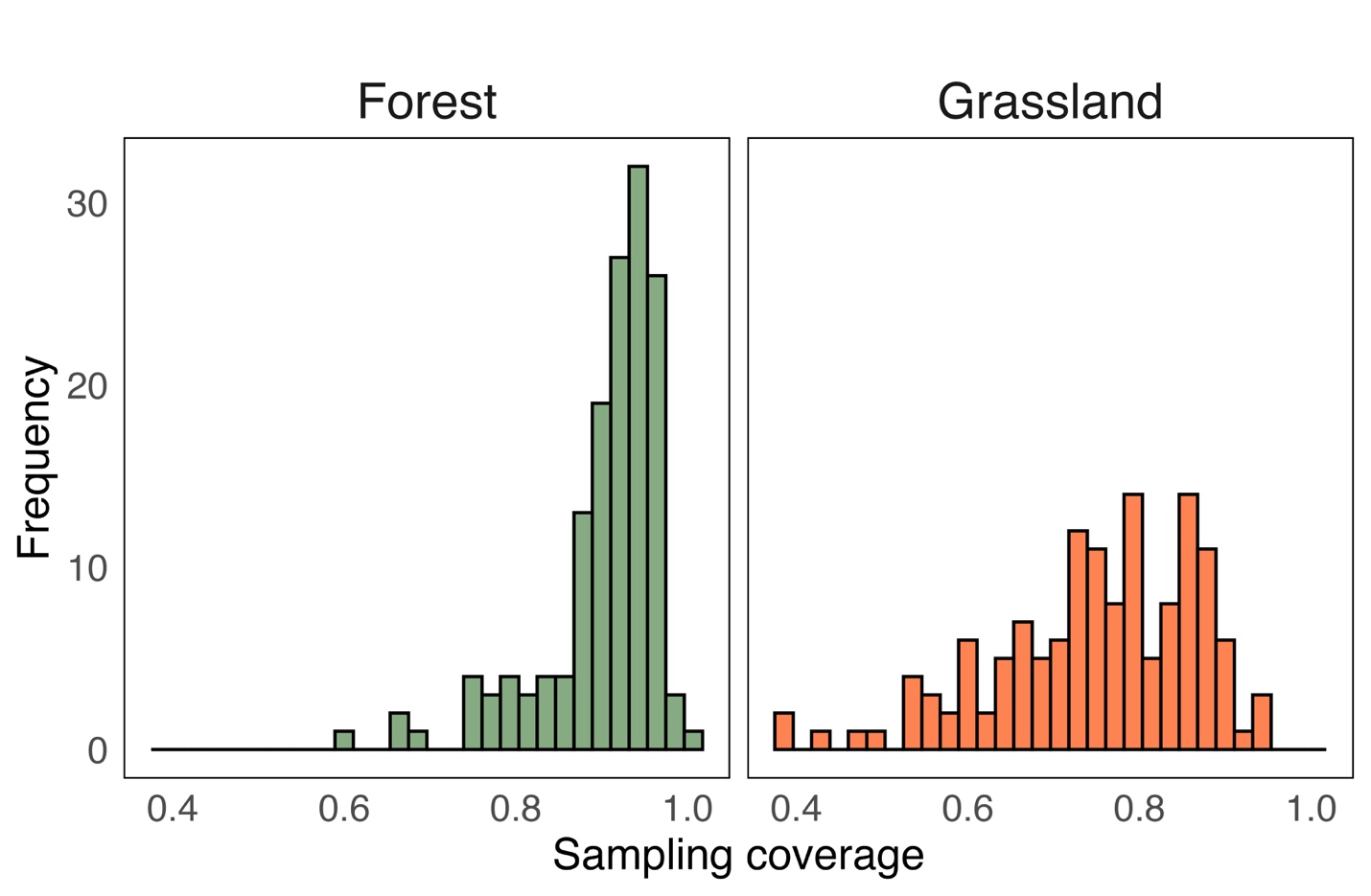


**Figure S8.** Histograms of sampling coverage for moth diversity assessments in forest and grassland habitats. Higher sampling values indicate more complete sampling of the moth community. The observed distributions highlight differences in sampling efficiency between habitats.


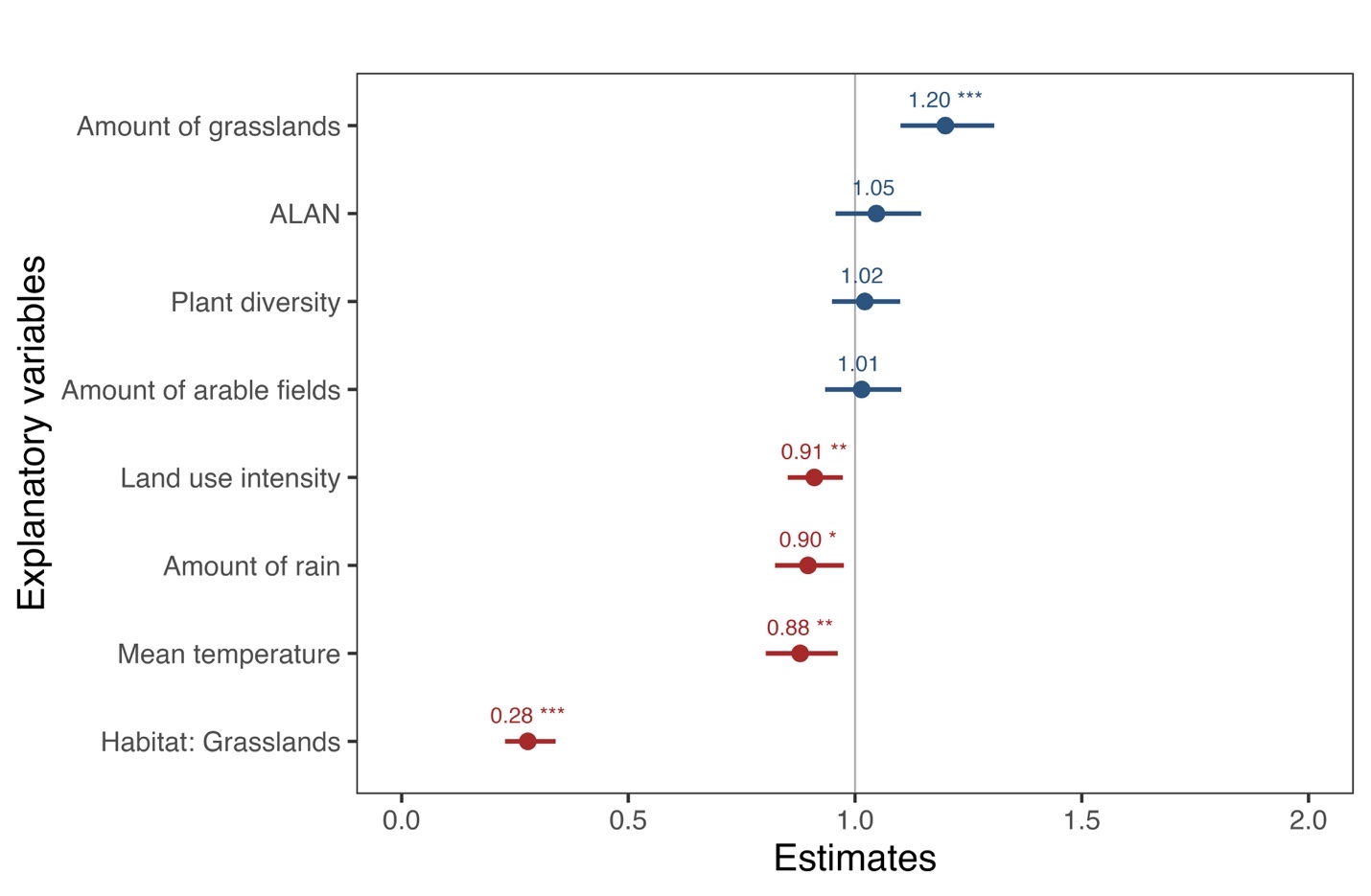


**Figure S9.** Standardized model estimates (±95% confidence intervals) for the effects of explanatory variables on sample coverage. Positive estimates (blue) indicate variables associated with increased coverage, while negative estimates (red) represent variables linked to reduced coverage. Significant effects are denoted by asterisks: *p* < 0.05 (*), *p* < 0.01 (**), and *p* < 0.001 (***).


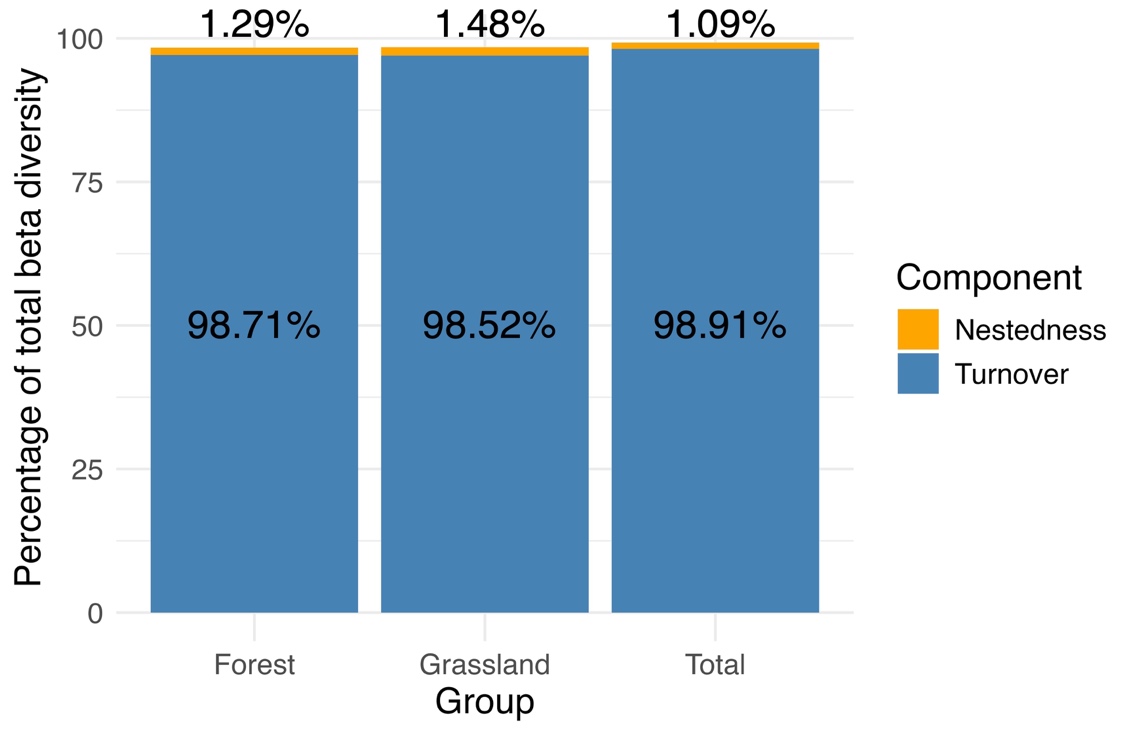


**Figure S10.** Partitioning of abundance-based multiple-site dissimilarity (using the *betapart* package, v.1.6; Baselga et al., 2023) revealed that turnover (balanced variation in abundance) overwhelmingly accounted for the vast majority of beta diversity (total, grassland, and forest; all >98%), while the contribution of nestedness (abundance gradients) was minimal (<2%). This pattern indicates that variation in moth community composition among plots is driven almost entirely by species replacement, rather than by sites being subsets of each other in terms of abundance. We quantified compositional dissimilarity among moth communities using abundance-based beta-diversity partitioning. Based on the site-by-species abundance matrix used as well for the MRM analyses, dissimilarity was partitioned into its turnover (balanced variation in abundance) and nestedness (abundance gradients) components following Baselga’s framework (Baselga et al. 2010). Specifically, we applied the *beta.multi.abund* function to the abundance matrix for the entire dataset, as well as separately for grassland and forest plots. This approach yields three metrics: total beta diversity, the proportion attributable to turnover, and the proportion attributable to nestedness. Percentages for each component were calculated as their contribution to total beta diversity.


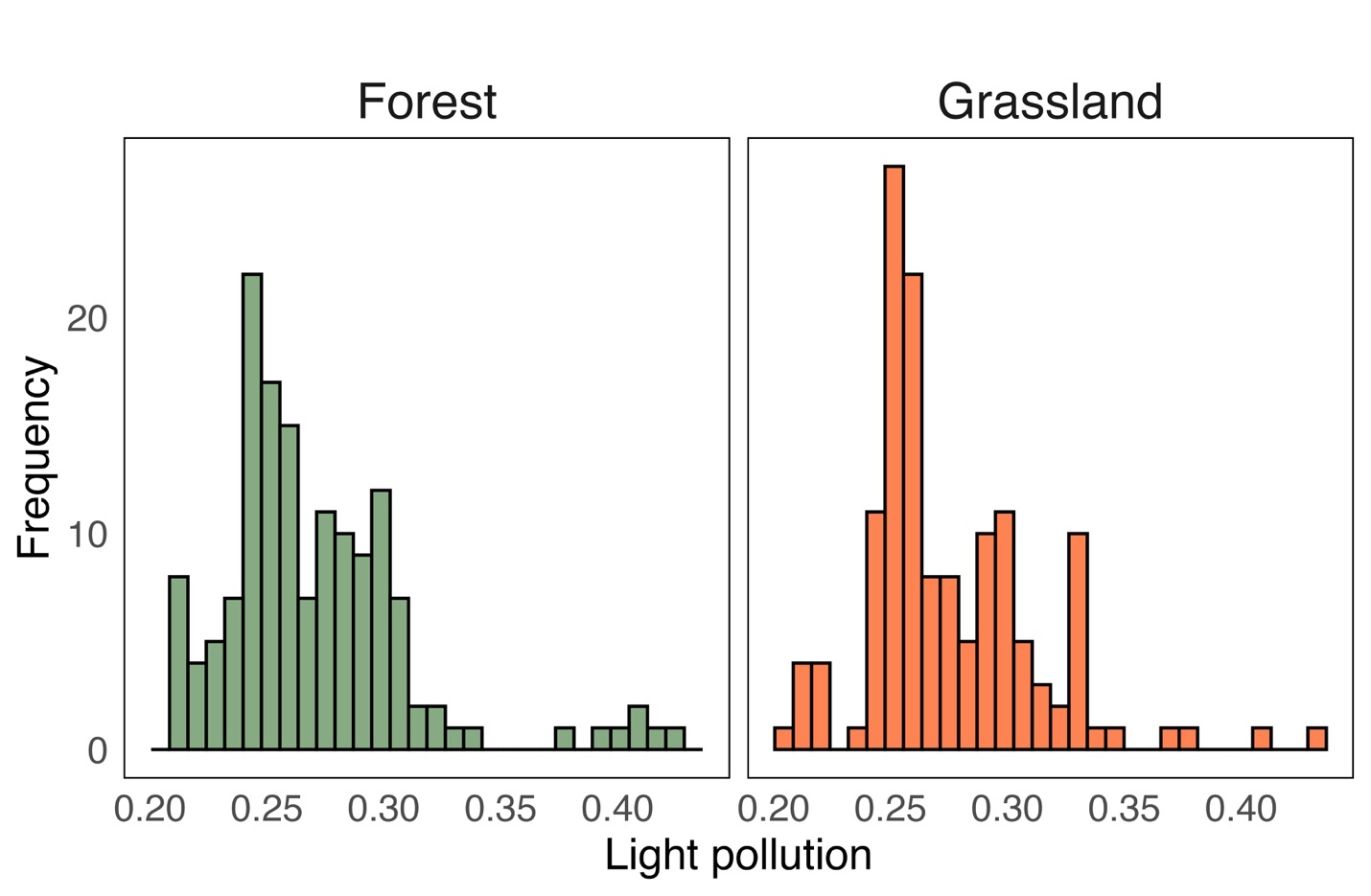


**Figure S11.** Distribution of artificial light at night (ALAN) intensity across study plots in two habitat types: forest (green) and grassland (orange). Values of light pollution (ALAN) represent zenith sky brightness (μcd/m²) derived from the World Atlas of Artificial Night Sky Brightness (Falchi et al., 2016) at a spatial resolution of 15 arcseconds. Histograms display the frequency of plots across the observed range of light pollution intensities.

**Additional discussion excluding vs including all data**

For diversity analyses, we accounted for sampling completeness based on coverage estimates for each plot. To ensure that diversity patterns were not biased by trap failures, we applied a conservative threshold of more than 10 individuals per plot. This cutoff was identified during the abundance analyses as the point at which trap failure no longer affected sampling completeness (Table S2). Low-abundance plots are more likely to reflect technical failures rather than true absence of moths. Despite applying this threshold, the high trapping success rate (97.3%) and the large dataset (71,388 individuals, 455 species) underscore the robustness of our sampling approach, ensuring that diversity patterns reflect ecological processes rather than sampling artifacts. This practice is widely accepted in ecological studies addressing imperfect detection in mobile insect taxa (Uhl et al., 2022). We argue that explicitly accounting for trap failure is essential when using automated methods of detection or attraction, an issue often overlooked in biodiversity research.

In addition to the main analyses (Figure 2, Table S5), we conducted a sensitivity analysis including all plots, regardless of moth abundance (Table S6). The main ecological patterns, such as the positive effects of temperature and plant diversity, and the negative effect of grassland habitat, remained consistent, supporting the robustness of our findings. However, minor differences emerged for variables with smaller or non-significant effects (e.g., land-use intensity, or rain), which showed slight variability between datasets. These differences were limited to species richness (q = 0) and Simpson diversity (q = 2), while Shannon diversity (q = 1) remained stable. For example, the negative effect of grassland on species richness (q = 0) became slightly weaker, and the positive effect of land-use intensity on species richness (q = 0) became marginally significant at 90% coverage. These shifts likely result from increased stochasticity introduced by plots with very low numbers of individuals, which inflate variability, particularly in evenness-weighted metrics like q = 2.

Both ecological and statistical reasoning support the applied threshold. While including all plots maximizes spatial coverage, in our case this involved only four additional plots, it risks introducing bias because captures of fewer than 10 individuals were strongly associated with trap failures (Table S2). Conversely, filtering improves data quality without compromising ecological inference when clear evidence of sampling bias exists (Magurran & Hill, 2011), a common trade-off in biodiversity studies. The high consistency between the main and sensitivity analyses confirms that our conclusions are not dependent on this filtering step. Nonetheless, we recognize that both approaches have strengths and limitations. Excluding poorly sampled plots prioritizes data quality and reduces sampling bias, whereas including them increases sample size but may dilute ecological signals with sampling noise. We recommend that future studies combine rigorous sampling protocols with complementary approaches, such as coverage-based rarefaction (Chao & Jost, 2012), to further mitigate the effects of imperfect detection in biodiversity assessments.

**References**

Baselga, A. (2010). Partitioning the turnover and nestedness components of beta diversity. *Global Ecology and Biogeography*, 19(1), 134–143.

Baselga, A., Orme, D., Villeger, S., De Bortoli, J., Leprieur, F., Logez, M., Martinez-Santalla, S., Martin-Devasa, R., Gomez-Rodriguez, C., & Crujeiras, R. (2023). betapart: Partitioning Beta Diversity into Turnover and Nestedness Components (R package version 1.6). <https://CRAN.R-project.org/package=betapart>

Chao, A., & L. Jost. (2012) Coverage-based rarefaction and extrapolation: standardizing samples by completeness rather than size. *Ecology*, 93(12), 2533–2547.

Magurran, A. E., & McGill, B. J. (Eds.). (2011). Biological diversity: Frontiers in measurement and assessment. Oxford University Press

Memmert, Jörg. (2023) Overview maps of all plots in the three regions of the Biodiversity Exploratories. Version 6. Biodiversity Exploratories Information System. Dataset ID= 31151. https://www.bexis.uni-jena.de.

Uhl, B., Wölfling, M., & Fiedler, K. (2022). Exploring the power of moth samples to reveal community patterns along shallow ecological gradients. *Ecological Entomology*, 47(3), 371-381.
